# Primary sensory cortices contain distinguishable spatial patterns of activity for each sense

**DOI:** 10.1038/ncomms2979

**Published:** 2013-06-11

**Authors:** M. Liang, A. Mouraux, L. Hu, G.D. Iannetti

**Affiliations:** 1 Department of Neuroscience, Physiology and Pharmacology, University College London, London WC1E 6BT, UK; 2 Institute of Neuroscience (IoNS), Université catholique de Louvain, Brussels B-1200, Belgium; 3 Key Laboratory of Cognition and Personality and School of Psychology, Southwest University, Chongqing 400715, China

## Abstract

Whether primary sensory cortices are essentially multisensory or whether they respond to only one sense is an emerging debate in neuroscience. Here we use a multivariate pattern analysis of functional magnetic resonance imaging data in humans to demonstrate that simple and isolated stimuli of one sense elicit distinguishable spatial patterns of neuronal responses, not only in their corresponding primary sensory cortex, but in other primary sensory cortices. These results indicate that primary sensory cortices, traditionally regarded as unisensory, contain unique signatures of other senses and, thereby, prompt a reconsideration of how sensory information is coded in the human brain.

## Abstract (web summary)

Human primary sensory cortices are traditionally regarded as being able to process only one sensory modality. Liang and colleagues use brain imaging to show that, as well as being processed in typically corresponding cortical areas, different sensory modalities are also processed in atypical cortical areas.

## Graphical abstract

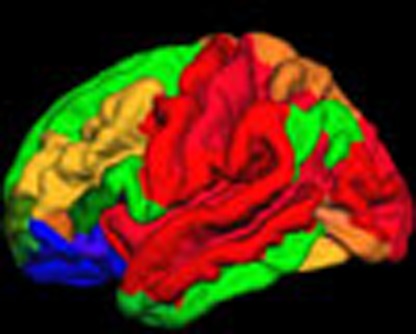

The human brain receives a continuous flow of information from different senses. Processing this information is key to perception and behaviour. However, how sensory information is hierarchically processed in the cerebral cortex has been very much debated in recent years. The traditional view is that information from different senses is initially processed in anatomically distinct, primary unisensory areas and subsequently converges onto higher-order multisensory areas^1^,^2^. This notion is based on early evidence, both in animals and humans, of anatomo-functional segregation between different unisensory areas, as well as between unisensory and multisensory areas. First, lesions limited to primary sensory cortices (PSCs) determine clearly unimodal sensory deficits^3^,^4^,^5^. Second, electrophysiological and functional neuroimaging studies report that sensory stimuli elicit activity in the primary sensory areas corresponding to the sensory modality of the eliciting stimulus, but not in other non-corresponding unisensory areas^6^,^7^,^8^,^9^. Third, tracing studies had demonstrated very few, if any, interconnections between primary somatosensory, auditory and visual cortices^10^.

An alternative hypothesis challenging this traditional view has been recently proposed—that these cortical areas, traditionally believed to be strictly unisensory, are instead multisensory^2^,^11^. There are two lines of evidence supporting this alternative possibility. First, a number of studies have shown that the responses elicited in unisensory cortices by corresponding sensory input can be modulated by concurrently applied non-corresponding sensory input^12^,^13^,^14^,^15^. For example, using high-resolution functional magnetic resonance imaging (fMRI) in macaque monkeys, it was observed that temporally coincident tactile stimuli enhance the activity elicited in or near the primary auditory cortex by auditory stimuli^15^. Second, other studies have showed that activity in PSCs can be elicited by stimuli belonging to a non-corresponding sensory modality, but only when these stimuli convey information related to the modality of the explored PSC, likely related to sensory imagery^16^,^17^,^18^. For example, visual stimuli conveying information related to the auditory modality (like, a silent videoclip of a barking dog or of a violin being played) have been shown to elicit activity in the auditory cortex^18^.

It is crucial to note that the two types of experiments detailed above do not provide unequivocal evidence that PSCs are essentially multisensory. Indeed, the observed multisensory effect either consist in a modulation of principal responses by concurrent stimuli of other modalities, or could result from stimulus-triggered sensory imagery within the modality corresponding to the PSC from which the responses are recorded.

Therefore, two key questions remain unresolved. First, can PSCs respond to stimuli of other senses when they are not temporally coincident with stimuli of the principal modality of the PSC from which the response is sampled, and/or when they do not trigger sensory imagery within that principal modality? Second, are such non-principal responses elicited in PSCs unique for each modality? In other words, are the responses elicited in a given primary sensory area (for example, V1) by stimuli belonging to two different and non-corresponding sensory modalities (for example, an auditory and a tactile stimulus) distinguishable?

Here, using a multivariate pattern analysis (MVPA) of fMRI signals in the human primary somatosensory (S1), auditory (A1) and visual cortex (V1), we examined the spatial patterns of the neural responses elicited by the presentation of simple and isolated tactile, painful, auditory and visual stimuli (Experiment 1), or tactile stimuli delivered to two different body locations and visual stimuli delivered to two different visual field locations (Experiment 2). We demonstrate that, in any explored PSC, the spatial pattern of the normalized fMRI responses elicited by each sensory stimulus of another modality is sufficiently distinct to allow a reliable classification of the stimulus modality (for example, discrimination between tactile and auditory stimuli using the fMRI responses sampled within V1). We further demonstrate that two stimuli of the same modality presented in different locations of the receptive surface also elicit distinguishable patterns of fMRI responses in non-corresponding PSCs (for example, discrimination between tactile stimuli delivered to two fingers using the fMRI responses sampled within V1). These findings indicate that transient and isolated stimuli of one sense elicit distinguishable spatial patterns of neural activity not only in their corresponding PSC but also in non-corresponding PSCs.

## Results

### PSCs encode the modality of non-corresponding stimuli

To avoid inducing responses related to multisensory integration and/or sensory imagery in PSCs, we delivered simple and isolated stimuli of four sensory modalities (tactile, painful, auditory and visual). Brain responses were sampled using 3T fMRI in 14 healthy participants, in four runs. Each run included an equal number of stimuli of each modality. Three anatomical masks corresponding to the PSCs (S1, A1 and V1) were defined in each participant using the Jülich probabilistic atlas^19^. MVPA^20^,^21^ was used to test the uniqueness of the spatial pattern of blood oxygen level-dependent (BOLD) activity elicited in each PSC by each type of sensory stimulus. It is important to note that in the present study the MVPA was performed on normalized BOLD fMRI signals. This ruled out the possibility that the MVPA classification was due to bulk differences in magnitude of the responses to different stimuli (owing, for example, to differences in stimulus saliency, arousal or attention) and, thus, ensured that it was necessarily due to differences in the fine-grained spatial distribution of the activity within the tested brain region. Also, the classification accuracy was not driven by differences in head motion between conditions (repeated-measures analysis of variance performed for each of the six motion parameters, in every subject: *P*_min_=0.39, *P*_max_=1.00, *P*_median_=0.97).

**Figure 1:**
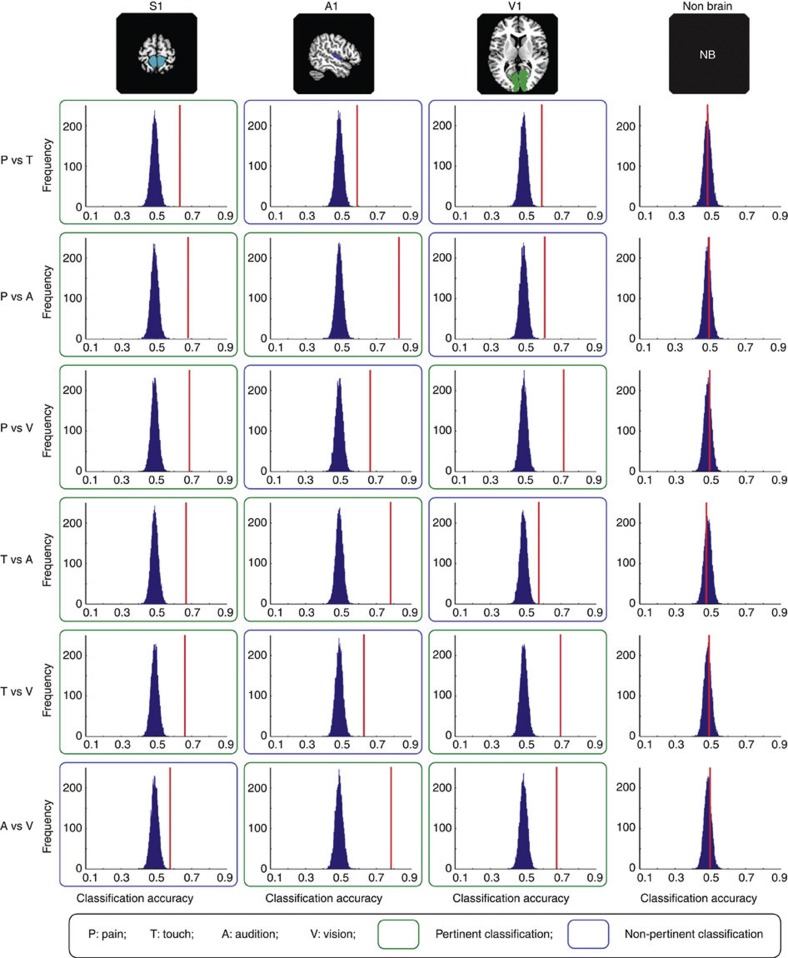
Classification accuracies and corresponding null distributions in Experiment 1. Null distributions were generated from 10,000 random permutations from data sets of 14 participants (see Methods for details). Classification accuracies are indicated by vertical red lines (see also Supplementary Fig. S1 and Table S1 for individual classification accuracies and statistical results). Each column represents a ROI defining a primary sensory cortex (S1, A1, V1 or a control, non-brain area). Each row represents a two-way classification. ‘Pertinent’ classifications (green frames) refer to the discrimination between two stimuli, one of which corresponds to the principal modality of the ROI. ‘Non-pertinent’ classifications refer to the discrimination between two stimuli, none of which corresponds to the principal modality of the ROI. Permutation tests (*n*=10,000) show that all classification accuracies are significantly greater than chance level (*P*<0.0001) except the accuracies from the non-brain control area (*P*=0.5, *P*=0.33, *P*=0.28, *P*=0.63, *P*=0.33, *P*=0.25 for ‘pain versus touch’, ‘pain versus audition’, ‘pain versus vision’, ‘touch versus audition’, ‘touch versus vision’ and ‘audition versus vision’, respectively).

In each of the three PSCs (S1, A1 and V1), six two-way classifications were performed: ‘pain versus touch’, ‘pain versus audition’, ‘pain versus vision’, ‘touch versus audition’, ‘touch versus vision’ and ‘audition versus vision’. The group-average accuracies of each classification task are shown in Fig. 1 (red vertical lines), Supplementary Fig. S1b (red horizontal lines) and Supplementary Table S1. The classification accuracies obtained in each individual participant are also shown in Supplementary Fig. S1b (coloured dots). A statistical *P*-value against the chance level (50% for two-way classifications) was determined for each classification and region of interest (ROI) by comparing the group-average accuracy with its corresponding null distribution generated by 10,000 random permutations.

The results of this analysis showed that the accuracy of each classification was significantly higher than chance level regardless of whether one of the two sensory modalities involved in the classification corresponded to the modality of the given PSC (all *P*<0.0001, that is, none out of 10,000 random permutations reached the actual classification accuracies obtained from correctly labelled data). This finding clearly indicates that the spatial distribution of the normalized fMRI responses elicited in each PSC by each type of sensory stimulus was sufficiently distinct to allow a reliable classification of its modality (for example, correct discrimination between tactile and auditory stimuli using the fMRI response in V1). When comparing the classification accuracies of pertinent and non-pertinent tasks, pertinent classifications showed significantly higher accuracies than non-pertinent classifications, in all ROIs (Supplementary Tables S2–S4). Above-chance-level classification accuracies were also achieved when performing MVPA using the BOLD signal from the PSCs of each hemisphere separately (Supplementary Table S5). Importantly, possible saliency-related differences in BOLD signal did not contribute to classification accuracy (S1: *r*=0.17, *P*=0.11; A1: *r*=0.07, *P*=0.55; V1: *r*=0.07, *P*=0.51; Spearman’s correlation between the difference in saliency ratings and the corresponding classification accuracy). An additional analysis performed by repeating the same MVPA procedure on a control region composed of voxels randomly selected outside the brain showed that the accuracies of all two-way classifications were always near chance level (Fig. 1 and Supplementary Fig. S1; Supplementary Table S1).

The MVPA results reported above were obtained using the second fMRI volume acquired 4–6 s after each stimulus onset, that is, the volume corresponding to the expected peak of the BOLD response (Supplementary Fig. S1) and, thus, most likely to contain stimulus-related information. To reveal the time-course of the classification accuracy, we repeated the same MVPA using the fMRI volumes acquired before and after stimulus onset, for each ROI (Supplementary Fig. S2). This analysis revealed that classification accuracies were near chance level before the stimulus, peaked at 4–6 s and returned to pre-stimulus baseline level after 13–15 s. This temporal profile is consistent with the known time-course of the BOLD fMRI response^22^, and confirms that the successful prediction obtained using the 2nd volume is truly based on stimulus-induced neural activity within PSCs.

**Figure 2:**
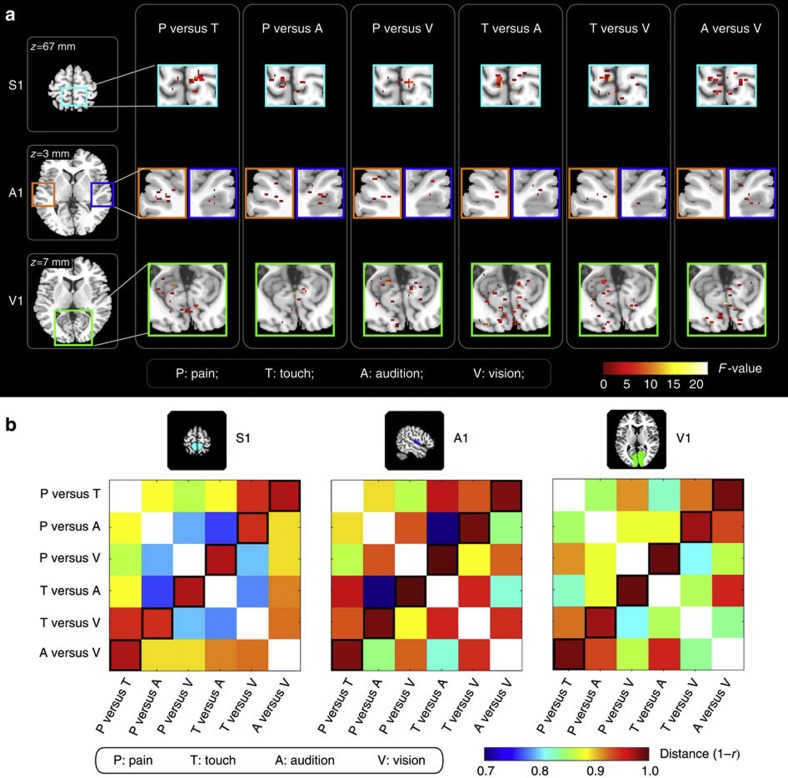
Group-level sensitivity maps and distance matrices in Experiment 1. (**a**) Group-level sensitivity maps obtained from 14 participants, showing the sparse spatial distribution of voxels significantly contributing to each classification task (*F*-test; *F*_(1,13)_>4.67, *P*<0.05). *F*-values are represented in colour and express the contribution of each voxel to each classification task after inter-subject variability was taken into account. (**b**) Group-level distance matrices between sensitivity maps of different classifications, in each ROI (S1, A1, V1). The values in the antidiagonal entries (bottom-to-top, left-to-right) show the maximal distance between the maps of classifications with non-overlapping sensory modalities (for example, ‘pain versus audition’ and ‘touch versus vision’).

To visualize the spatial distribution of voxels that contributed most to the successful classifications, we computed, for each participant and classification task, sensitivity maps showing the weights obtained by linear support vector machine (SVM) (Fig. 2a and Supplementary Fig. S3). These maps revealed that the voxels contributing most to the classification accuracy were scattered within each PSC and, most importantly, that their spatial distributions were different for the different two-way classifications (Fig. 2a). We also used conventional general linear model (GLM) analysis to calculate the beta-values of the voxels contributing most to the classifications, and thus examine whether the classification accuracies were determined by BOLD signal increases (positive beta-values), BOLD signal decreases (negative beta-values) or a mixture of the two. This analysis showed that, regardless of which two sensory modalities were discriminated, the contributing voxels in a given ROI always responded more strongly to their corresponding sensory modality than to their non-corresponding modalities (Supplementary Fig. S4). Furthermore, the direction of the BOLD responses elicited by stimuli of non-corresponding modalities was mixed, that is, the classification accuracy was determined both by voxels with positive BOLD responses and voxels with negative BOLD responses (Supplementary Figs S5,S6). Taken together, these observations indicate that, within each PSC, the information of each stimulus modality is distinguishable at spatially distributed pattern level, and explains why such spatial differences cannot be easily detected by conventional mass-univariate analysis^21^.

To further characterize the spatial distribution of the voxels contributing to the different classifications, we calculated, for each PSC, a distance matrix describing the dissimilarity of the group-level sensitivity maps between two different two-way classification tasks. A higher distance indicates more different spatial distributions of contributing voxels between two classifications. This dissimilarity analysis revealed that the spatial locations of voxels contributing to classifications of non-overlapping pairs of sensory modalities (for example, ‘pain versus vision’ and ‘touch versus audition’ have non-overlapping sensory modalities, whereas ‘pain versus vision’ and ‘touch versus vision’ have an overlapping modality, that is, vision) were the most dissimilar (Fig. 2b). This finding further confirms that each sensory modality elicits a distinguishable spatial pattern of neural activities in each PSC.

**Figure 3:**
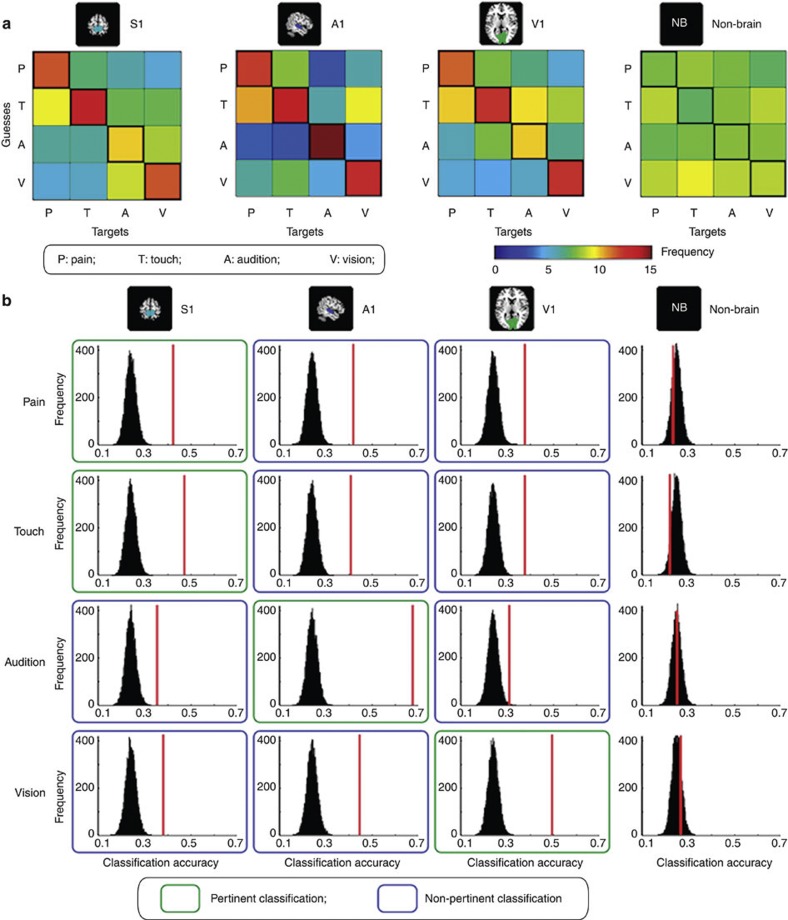
Classification accuracies of four-way classifications in Experiment 1. (**a**) Group-average confusion matrices for all ROIs. The colour of each entry indicates the number of guesses made by the classifier for each sensory modality (*y* axis, guesses) against the actual modality of the eliciting stimulus (*x* axis, targets). Correct guesses are thus located in the top-to-bottom, left-to-right diagonal line. For all ROIs (except the control, non-brain ROI) confusion matrices exhibited a clear diagonal structure, indicating that the number of correct guesses was greater than chance level. (**b**) Group-average classification accuracies (vertical red lines) and corresponding null distributions generated from 10,000 random permutations from 14 participants. Each column represents a ROI. Each row represents the accuracy in predicting a given sensory modality (that is, the diagonal of the confusion matrix). ‘Pertinent’ classifications (green frames) refer to the prediction of target stimuli whose modality corresponds to the modality of the ROI. ‘Non-pertinent’ classifications refer to the prediction of target stimuli whose modality does not correspond to the modality of the ROI. Permutation tests (*n*=10,000) show that all classification accuracies are significantly greater than chance level (*P*=0.0013 for predicting ‘audition’ in V1; *P*<0.0001 for all the rest) except the accuracies from the non-brain area (*P*=0.75, *P*=0.91, *P*=0.44, *P*=0.18 for predicting ‘pain’, ‘touch’, ‘audition’ and ‘vision’, respectively).

To test the ability of the classifier to predict the modality of the eliciting stimulus out of all four possible sensory modalities, a four-way classification was performed in each PSC of each participant. The accuracies of the four-way classifications are summarized as a 4 × 4 confusion matrix with each entry indicating the number of guesses made by the classifier for each stimulus modality (Fig. 3a). A confusion matrix of a successful classifier will display higher values in the top-to-bottom, left-to-right diagonal entries (that is, entries representing correct guesses). Confusion matrices of all PSCs revealed a clear diagonal pattern, indicating good classification accuracy (Fig. 3a). Permutation testing (*n*=10,000; Fig. 3b, Supplementary Table S6) confirmed that the classification accuracies were always significantly greater than chance level (25%), even when the modality of the target stimuli did not correspond to that of the PSC (all *P*<0.0001, meaning that none out of 10,000 random permutations reached the actual classification accuracies obtained from correctly labelled data, except *P*=0.0013 when predicting auditory stimuli using BOLD signals in V1). In contrast, all classification accuracies obtained from the non-brain control region were around chance level (Fig. 3a, Supplementary Table S6). These results show that, even for four-way classifications, the distributed spatial pattern of BOLD responses in any given primary sensory area can reliably predict the modality of the eliciting stimulus, regardless of whether its sensory modality matches that of the primary sensory area.

### Modality coding in PSCs is not determined by edge voxels

To test whether the successful predictions of the seven non-pertinent classifications could be merely determined by the fMRI signal obtained from voxels included in the PSC ROIs, but possibly belonging to neighbouring higher-level areas, in Control analysis A, we repeated the MVPA on two different sets of ROIs: smaller, eroded ROIs, generated by removing the voxels constituting the outer layer of the original ROIs, and ROIs manually defined based on anatomical landmarks. This control analysis yielded essentially the same results as in the main analysis (Supplementary Figs S7,S8). Therefore, although these results do not allow us to conclude that the core of the PSCs has a definite role in discriminating different sensory modalities, they rule out the possibility that voxels located in the peripheral part of the ROIs, possibly sampling neural activity of neighbouring higher-order areas, determined the successful predictions in the non-pertinent classification tasks.

### Modality coding is observed in some other brain regions

To test whether the ability of discriminating between all sensory modalities is pervasive across the whole brain, in Control analysis B we parcellated the brain into 116 regions based on the AAL atlas^23^ and repeated the same MVPA on each of these regions. The results showed that only a subset of all brain regions appeared to contain information allowing discrimination of the sensory modality of the eliciting stimulus (Supplementary Fig. S9 and Table S7).

### PSCs also encode the location of non-corresponding stimuli

In Experiment 1, the stimuli of each sensory modality were identical in their spatial location, intensity and temporal profile. Therefore, it was unclear whether different stimulus features besides modality can also be discriminated using BOLD signals in non-corresponding PSCs. To address this question, in Experiment 2 we recorded the fMRI responses to somatosensory stimuli (10-Hz innocuous electrical pulses) delivered to either the index finger (‘Touch 1’) or the little finger (‘Touch 2’) of the right hand, and to visual stimuli (10-Hz pattern reversal wedge-shaped checkerboards of 90° arc) presented in either the upper-right (‘Vision 1’) or the lower-right (‘Vision 2’) visual field (Supplementary Fig. S10). This allowed us to test whether BOLD signals in PSCs allow discriminating (1) between two different non-corresponding stimuli of the same modality, and (2) between non-corresponding modalities even when the responses to two different stimuli of the same modality (for example, ‘Touch 1’ and ‘Touch 2’) are pooled together.

**Figure 4:**
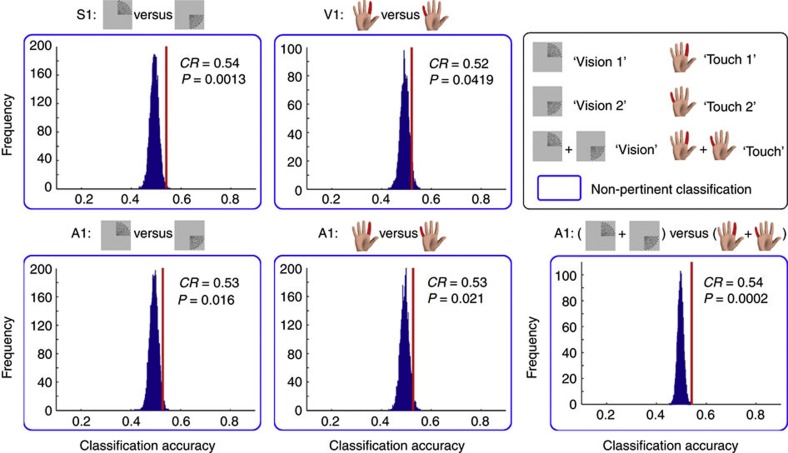
Classification accuracies and null distributions of non-pertinent classification tasks in Experiment 2. Group-average classification accuracies (CR, correct rate) and corresponding *P*-values (obtained from 10,000 permutations) were obtained from 14 participants and are shown in the top-right corner of each inset. ‘Non-pertinent’ classifications refer to the discrimination between two stimuli, none of which corresponds to the principal modality of the ROI. ‘Vision 1’: visual stimuli presented on the upper-right visual field, ‘Vision 2’: visual stimuli presented on the lower-right visual field, ‘Touch 1’: somatosensory stimuli delivered to the right index finger, ‘Touch 2’: somatosensory stimuli delivered to the right little finger. ‘Touch’: pooled ‘Touch 1’ and ‘Touch 2’ stimuli, ‘Vision’: pooled ‘Vision 1’ and ‘Vision 2’ stimuli.

The MVPA yielded two results. First, BOLD signals allowed higher-than-chance discrimination between two different non-corresponding stimuli (‘Vision 1’ versus ‘Vision 2’ in S1: *P*=0.0013; ‘Touch 1’ versus ‘Touch 2’ in V1: *P*=0.0419; ‘Touch 1’ versus ‘Touch 2’, and ‘Vision 1’ versus ‘Vision 2’ in A1: *P*=0.021 and 0.016, respectively). Second, BOLD signals in A1 allowed higher-than-chance discrimination between ‘Touch’ and ‘Vision’ even when different stimuli of the same modality were pooled together (*P*=0.0002). Classification accuracies and permutation testing are shown in Fig. 4. These results indicate that BOLD signals elicited by non-corresponding stimuli in PSCs allow discriminating not only the modality of the eliciting stimuli, but also the spatial location of stimuli belonging to the same modality (although, in this case, classification accuracies, albeit always significantly higher than chance level, were reduced).

## Discussion

In striking contrast with what is observed when analysing the data with traditional voxel-by-voxel univariate analysis of signal amplitude, our results demonstrate that transient and isolated stimuli of a given sensory modality elicit distinguishable spatial patterns of neural activity not only in their corresponding primary sensory areas, but also in non-corresponding primary sensory areas (Experiment 1, Fig. 1). Importantly, when two stimuli of the same modality are presented in different spatial locations, these stimuli also elicit distinguishable patterns of BOLD signals in non-corresponding PSCs (Experiment 2, Fig. 4). The scattered patterns revealed by the sensitivity maps (Fig. 2a and Supplementary Fig. S3), and the fact that the responses to non-corresponding sensory input constitutes a mixture of BOLD signal increases and decreases (Supplementary Figs S5,S6) may explain why these responses are unlikely to be detectable using conventional mass-univariate GLM analysis. Indeed, spatial smoothing is commonly applied to fMRI time-series to increase the signal-to-noise ratio. Furthermore, cluster thresholding, a commonly used method to control false-positive rate in mass-univariate analysis, implies consistent activation across neighbouring voxels.

Our results are very different from the few previous experimental results showing (i) that the activity elicited within a given PSC by its corresponding sensory stimuli can be modulated by the concomitant presentation of non-corresponding sensory stimuli (for example, when the activity elicited by visual stimuli in V1 is modulated by concomitant tactile input)^12^,^13^,^14^ or (ii), that complex unisensory stimuli conveying information pertaining to another sensory modality can elicit activity within the PSC corresponding to that other sensory modality (for example, silent videoclips of a barking dog eliciting activity in A1)^16^,^17^,^18^,^24^. Indeed, we used stimuli that were simple (that is, without any semantic content likely to trigger imagery in another sensory modality—although we cannot completely rule out the possibility that sensory imagery contributed to our finding) and presented in isolation (that is, without concomitant stimuli of another modality) to show that sensory input of any given modality elicits, *per se*, a characteristic pattern of activation in non-corresponding PSCs. Therefore, our finding suggests that the non-principal responses in PSCs are more fundamental than previously suggested by, for example, the observation that A1 can be activated by viewing silent lipreading but not by viewing nonlinguistic facial movements^16^. A recent study^24^ reported that early responses in the auditory cortex contained information about visual stimuli only when these stimuli were contextually relevant to the auditory system. Although these studies^16^,^24^ suggest an important role of imagery or contextual information in the multisensory interactions observed in unisensory cortices, they do not exclude the possibility that spatially distinguishable responses can be induced in PSCs by stimuli of non-corresponding modalities even when appropriate contextual information is absent. Our results provide compelling evidence that spatially distinct responses are elicited in PSCs by (1) stimuli of different non-corresponding sensory modalities and (2) stimuli of the same non-corresponding modality presented at different spatial locations. Most importantly, the fact that these patterns of activation are spatially distinct raises the possibility that sensory inputs belonging to different modalities activate distinct populations of neurons in each unisensory area.

The distinguishable patterns of neural activity in non-principal PSCs are likely to reflect cortico-cortical feedback projections from higher-order multisensory areas^25^,^26^,^27^ and/or feedforward projections from principal primary sensory areas or subcortical structures^28^,^29^,^30^. We suggest two possible reasons for this widespread distribution of sensory information in multiple PSCs.

First, the responses to non-corresponding sensory input could underlie processes involved in multisensory integration. Indeed, these responses could modulate the state of neurons in PSCs and, thereby, influence the processing of possibly concomitant corresponding sensory input. The observation that responses to non-principal sensory input were elicited by isolated unimodal stimuli (that is, in the absence of concomitant sensory input belonging to the principal sensory modality) does not necessarily argue against the hypothesis that the observed responses in unisensory areas might ultimately serve the function of facilitating multisensory integration. Indeed, it has been shown that unimodal somatosensory stimuli reset the phase of ongoing neural oscillations in A1 of awake macaques, possibly determining crossmodal effects (enhancement or suppression) on the responses elicited by concomitant auditory stimuli: depending on whether concomitant auditory input arrives during a high-excitability or a low-excitability phase, auditory responses would be enhanced or suppressed^31^.

Second, the distinguishable neural signature of each sensory modality observed in non-principal sensory areas could reflect a reduction or active inhibition of tonically active neurons, which may enhance the contrast between neural activities in principal and non-principal sensory areas^32^,^33^. Interestingly, a recent study performed in mice showed that the activation of A1 by a noise burst elicits hyperpolarization in the supra- and infragranular layers of V1 through cortico-cortical inputs that activate an inhibitory subcircuit originating in the deep layers of V1. This sound-driven local GABAergic inhibition on V1 resulted in reduced visually driven synaptic and spike responses upon bimodal audio-visual stimulation, thus suggesting that auditory cortex activation by salient auditory stimuli demotes the processing of potentially distracting visual stimuli within the visual cortex^33^. Building on this hypothesis, the distinguishable spatial patterns of non-principal responses observed in the present study suggests that, within each PSC, distinct local circuits are recruited by different non-corresponding sensory modalities. Furthermore, although electrophysiological studies in animals have suggested that, within PSCs, the number of neurons responding to non-corresponding sensory input is very limited^33^,^34^, our results suggest the opposite: that non-corresponding sensory stimuli activate a relatively large population of neurons, sufficient to elicit a detectable BOLD signal at the macro-scale level of fMRI data.

A top–down, attentional modulation of the neural activity in PSCs is not a likely explanation for the correct classification of the responses elicited by non-principal stimuli, because the attentional effects triggered by stimuli of different sensory modalities are not expected to modulate the activity within non-corresponding PSCs in a spatially distinct manner^35^,^36^,^37^. Indeed, a number of studies have shown that stimuli of different modalities activate a single supramodal attentional control network^35^,^36^, and it is unlikely that its possible top–down modulatory effect on PSCs is spatially different for different sensory modalities. The lack of correlation between the differences in subjective ratings of stimulus saliency and two-way classification accuracies also suggests that these were not driven by differences in attentional re-orientation or arousal. Furthermore, the MVPA classification was performed using normalized BOLD signals and, hence, correct classification of non-principal responses was not due to differences in the mean amplitude of the signal within each ROI but, instead, necessarily resulted from the fact that each of the different non-principal stimuli elicited a spatially distinct pattern of BOLD activity within each ROI. For the same reason, it seems unlikely that non-neural hemodynamic effects such as ‘blood stealing’^38^ could have contributed to the correct classification of non-pertinent responses. Indeed, not only have such hemodynamic effects been shown only in neighbouring areas sharing the same vasculature^38^, but also, should such long-distance effects occur, they could not explain the observation that touch and pain elicited spatially distinct BOLD signals in A1 and V1, given that the bulk of their BOLD responses are spatially indistinguishable, not only in S1 but also in the entire brain^39^.

Finally, Control analysis B showed that the ability to discriminate the sensory modality of the eliciting stimulus is not pervasive across all brain regions, albeit not unique to the PSCs. Indeed, only 24% of the 116 brain regions provided accurate responses in all six classification tasks (Supplementary Fig. S9 and Table S7). These regions included both unisensory and multisensory areas, particularly in the parietal, temporal and occipital lobes (Supplementary Fig. S9).

In summary, our findings provide a compelling answer to the ongoing debate about the extent of the multisensory nature of the neocortex^2^,^11^, demonstrating that even PSCs are essentially multisensory in nature. Crucially, the spatial patterns elicited by non-corresponding sensory input were distinguishable between different senses, suggesting that each sense elicits distinguishable spatial patterns of neural activities within each PSC. Importantly, these results do not argue against the notion that sensory inputs belonging to different sensory modalities are preferentially processed within their corresponding PSCs, and do not necessarily imply that PSCs are causally involved in the perception and sensory processing of non-corresponding sensory input. Rather, our results emphasize that PSCs do not solely respond to sensory input of their own principal modality. Following the present results, an intriguing question that needs to be addressed to understand the functional significance of these non-principal responses is whether they encode additional information besides the modality and spatial location of the applied stimulus, such as its intensity and frequency.

## Methods

### Sensory stimuli and design of Experiment 1

Functional MRI data were collected from 14 healthy participants who gave written informed consent and the experimental procedures were approved by the Oxford Central University Research Ethics Committee. Participants received stimuli of four sensory modalities: touch (transcutaneous electrical pulses over the superficial peroneal nerve), pain (laser pulses delivered on the foot dorsum), vision (a bright white disk presented above the right foot) and audition (right-lateralized 800 Hz tones delivered through pneumatic earphones). 3T fMRI data were acquired in a single session divided in four runs. Each run consisted of a stimulation period of 32 stimuli (8 stimuli/modality) pseudo-randomly delivered (inter-stimulus interval 10–19 s, <3 consecutive stimuli of the same modality), followed by a rating period of ~2 min during which participants rated the saliency of each stimulus type using a visual scale^40^,^41^. Saliency rating were not different across modalities (repeated-measures analysis of variance: *F*_(3,39)_=0.75, *P*=0.53). Detailed information can be found in Supplementary Methods.

### Regions of interest selection

The Jülich probabilistic histological atlas^19^ was used to define three anatomical regions of interest (ROIs) including bilateral primary somatosensory (S1; BA 3a/3b), auditory (A1; BA 41) and visual (V1; BA 17) cortices. Each ROI was constructed by binarising the corresponding probability volumes thresholded at *P*>0.5. The ROIs were then transformed into each participant’s high-resolution structural space. For each participant, the boundaries of ROIs defining S1 were trimmed to include only the mesial hemispheric wall (that is, the putative foot representation area of S1)^42^. Finally, all ROIs were transformed into each participant’s low-resolution functional space. These ROIs were the same as those used in our previous study^39^. The anatomical locations of these ROIs, together with their respective average BOLD responses are shown in Supplementary Fig. S1. An additional ROI was defined by randomly selecting voxels located outside the brain of each subject. The number of voxels included in this control ROI was made equal to the average number of voxels of the ROIs defining S1, A1 and V1.

### Multivariate pattern analysis

MVPA is a machine learning technique that uses a pattern classifier^21^,^43^,^44^ to identify the representational content of the neural responses elicited by different stimuli (in our case, stimuli belonging to four sensory modalities, based on the spatial pattern of the BOLD fMRI signal changes elicited by different stimuli). A brief introduction to MVPA, highlighting the key differences compared with conventional mass-univariate analyses is provided in Supplementary Methods.

After motion correction, linear detrending and normalization (Supplementary Methods), fMRI data were analysed using the PyMVPA software package^45^, in combination with LibSVM’s implementation of the linear SVM (www.csie.ntu.edu.tw/~cjlin/libsvm). A ‘leave-one-run-out’ cross-validation approach was employed to train and test the classifier. Considering that the TR was 3 s and the ISI was 10–19 s, at least three brain volumes were acquired after the onset of each stimulus. We used the 2nd volume after each stimulus onset (that is, the volume acquired at 4–6 s) for the MVPA in the main analysis, as this volume contains the peak of the BOLD signal elicited by each stimulus (Supplementary Fig. S1), and is thus the most likely to contain stimulus-related information. In addition, to reveal the time-course of the classification accuracies in each ROI, the same MVPA was also performed using the volume immediately before or at the onset of each stimulus (approximately −2 to 0 s) as well as the 1st (1–3 s), 3rd (7–9 s), 4th (10–12 s), 5th (13–15 s) and 6th (16–18 s) volumes (Supplementary Fig. S2).

For each ROI and subject, we performed all possible two-way classifications (that is, ‘pain versus touch’, ‘pain versus audition’, ‘pain versus vision’, ‘touch versus audition’, ‘touch versus vision’ and ‘audition versus vision’). A ‘leave-one-run-out’ cross-validation approach was employed to train and test the classifier algorithm: in each cross-validation step, the classifier was trained on three fMRI runs and tested on the fourth fMRI run. This procedure was repeated four times, using each time a different run as test data set. In each cross-validation step, classifier performance was calculated as the classification accuracy, that is, the number of correct guesses divided by the number of test trials. The overall performance for each classification task was obtained by averaging the classification accuracy obtained in each of the four cross-validation steps.

We also created sensitivity maps for each two-way classification tasks and ROI, in each participant. In these maps, the value of any given voxel represents its linear SVM weight. This value indicates the contribution of each voxel to the classifier’s accuracy in predicting the modality of the eliciting stimulus. Therefore, sensitivity maps can reveal which voxels within a given ROI provide greater contributions to each classification task.

For each ROI and participant, we also performed a four-way classification (that is, predicting the sensory modality of the stimuli eliciting the fMRI response out of four possible sensory modalities). This was achieved by first performing a binary SVM classification on each of the six category pairs, and then determining the final result of the multiclass classification by the sensory modality that was predicted more times among the six binary classifications. Similarly, a ‘leave-one-run-out’ cross-validation approach was employed. Such four-way classification yields, for each ROI and subject, a 4 × 4 confusion matrix, with each entry indicating the number of guesses made by the classifier for each stimulus modality. Individual confusion matrices were averaged across subjects, to obtain a group-level confusion matrix for each ROI.

### Statistical analysis

To test whether the accuracy of the classifier was higher than chance level (that is, 0.5 for two-way classifications and 0.25 for four-way classifications), we used permutation testing (*n*=10,000) performed for both training and testing data sets (similar results were obtained when permuting the training data set only), as detailed in Supplementary Methods.

In addition, in each ROI, we tested whether accuracies in pertinent classification tasks (that is, tasks in which one of the two sensory modalities corresponded to that of the tested PSC) were higher than in non-pertinent classification tasks (that is, tasks in which none of the sensory modalities corresponded to that of the tested PSC), using the paired Wilcoxon signed-rank test.

To test whether possible differences in BOLD signal related to saliency could have contributed to the classification accuracy, for each given two-way classification task we calculated the difference in saliency ratings between the two sensory modalities of that task, and then performed a Spearman’s correlation analysis between the difference in saliency ratings and the corresponding classification accuracy.

To obtain statistical, group-level sensitivity maps showing the differential contribution of the voxels composing each ROIs to each two-way classifications, single-subject maps (based on SVM weights) for each classification task and ROI were firstly transformed into standard MNI space. Then, the value of each voxel was tested against zero using an *F*-test, to determine which voxels had significant non-zero weights (that is, which voxels significantly contributed to the accuracy of the classification) across subjects (*F*_(1,13)_>4.67, *P*<0.05). In other words, the resulting *F*-maps can be considered as a group-level measure of contribution of each voxel after inter-subject variability is taken into account. Single-subject sensitivity maps of all non-pertinent classification in their individual, normalized anatomical space are shown in Supplementary Fig. S3.

To further characterize the relationship between sensitivity maps of different two-way classification tasks, we calculated, for each ROI, a distance matrix describing the dissimilarity of sensitivity maps between two different two-way classification tasks. This distance was defined as 1−*r*, where *r* is correlation coefficient between the two group-level sensitivity maps.

Finally, we also performed a conventional univariate GLM analysis on each voxel of the thresholded sensitivity maps of each ROI and subject, to test whether the voxels contributing most to the classification sampled BOLD signal increases (positive beta-values), decreases (negative beta-values) or a mixture of increases and decreases (Supplementary Figs S4–S6).

### Control analyses

In Control analysis A, we tested whether the voxels located on the outer layer of the ROIs (that is, voxels possibly reflecting neural activity of neighbouring higher-order areas) gave a determinant contribution to the successful predictions in the non-pertinent classification tasks by repeating the MVPA analysis on eroded ROIs and hand-drawn ROIs. In Control analysis B, we investigated whether the MVPA results obtained from PSCs could also be observed in other brain regions. Details of the control analyses are provided in Supplementary Methods.

### Sensory stimuli and design of Experiment 2

3T fMRI data were collected from a different group of 14 healthy participants who gave written informed consent, and the experimental procedures were approved by the Southwest University Ethics Committee. Participants received two different somatosensory stimuli and two different visual stimuli. Somatosensory stimuli were electrical pulses delivered to either the index finger (‘Touch 1’) or the little finger (‘Touch 2’) of the right hand. Visual stimuli were 10-Hz pattern reversal wedge-shaped checkerboards delivered to either the upper-right (‘Vision 1’) or the lower-right (‘Vision 2’) visual field. The same parameters of Experiment 1 were used for image acquisition and data analysis. Detailed information is provided in Supplementary Methods.

## Author contributions

M.L., A.M. and G.D.I. conceived and designed the study. A.M., G.D.I. and L.H. collected the data. M.L. analysed the data. M.L., A.M., L.H. and G.D.I. discussed the results and wrote the paper.

## Additional information

**How to cite this article:** Liang, M. *et al*. Primary sensory cortices contain distinguishable spatial patterns of activity for each sense. *Nat. Commun.* 4:1979 doi: 10.1038/ncomms2979 (2013).

## Supplementary Material

Supplementary Information Supplementary Figures S1-S10, Supplementary Tables S1-S7, Supplementary Methods and Supplementary References
