# Supplementary material for: Primary sensory cortices contain distinguishable spatial patterns of activity for each sense
Source: Nat Commun. 2013 Jun 11;4:1979. doi: 10.1038/ncomms2979 (PMC3709474; doi:10.1038/ncomms2979)
Supplement: Supplementary Information — Supplementary Figures S1-S10, Supplementary Tables S1-S7, Supplementary Methods and Supplementary References [file ncomms2979-s1.pdf]

SUPPLEMENTARY INFORMATION

for

Primary Sensory Cortices Contain Distinguishable Spatial Patterns of Activity for Each Sense

M Liang, A Mouraux, L Hu, GD Iannetti

Supplementary Information includes:

Supplementary Figures S1-S10

Supplementary Tables S1-S7

Supplementary Methods

Supplementary References

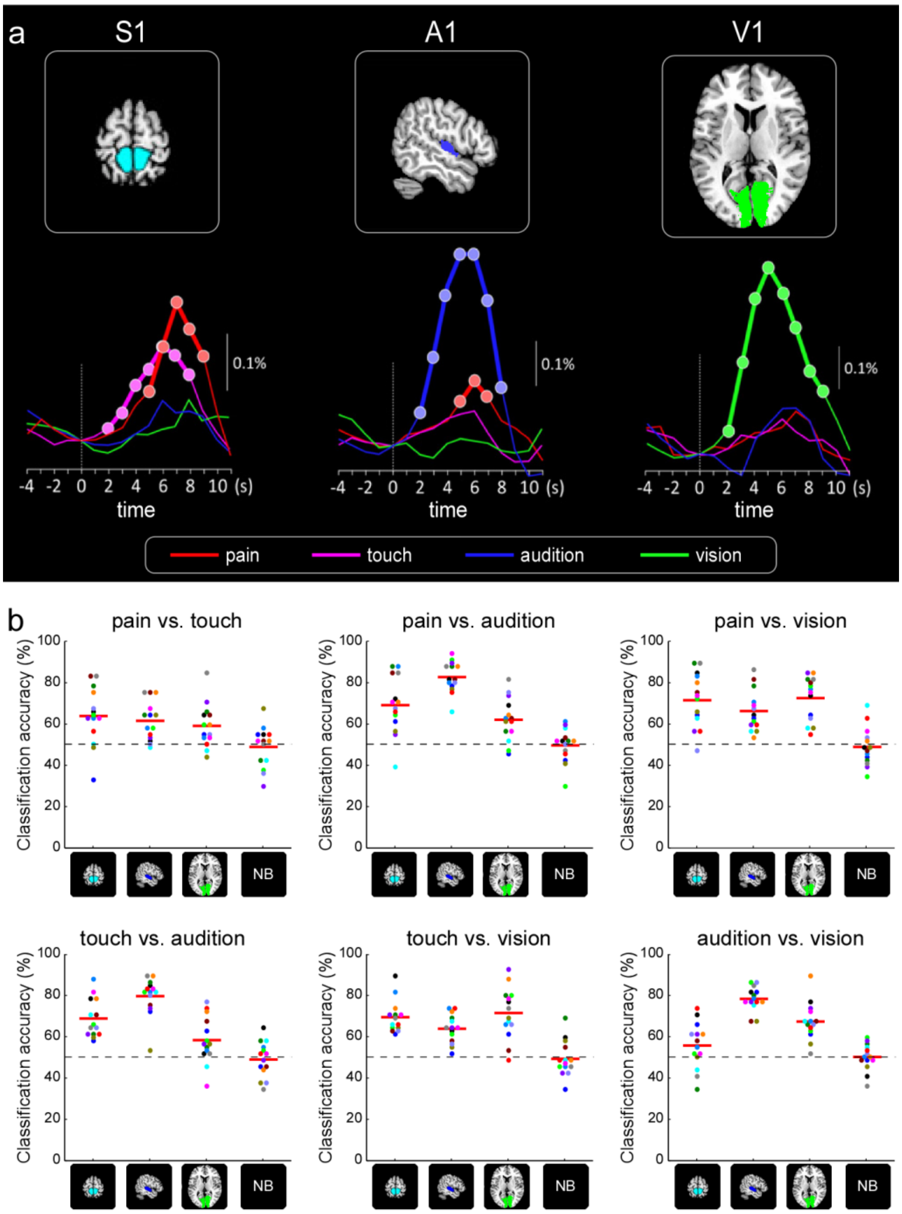

Supplementary Figure S1. Location of ROIs, their average BOLD time-courses and single-subject classification accuracies. (**a**) Upper panel: primary somatosensory (S1; cyan), auditory (A1, blue) and visual (V1, green) cortices defined based on the Jülich probabilistic histological atlas (see Methods). Lower panel: group-level average raw BOLD-signal time courses, relative to the onset of painful (red), tactile (purple), auditory (blue) and visual (green) stimuli. The vertical dashed lines correspond to stimulus onset. x axis: time (s); y axis: % signal change averaged across all voxels of each ROI. Time intervals showing a significant signal increase relative to baseline are highlighted by coloured disks and thick segments. Data are adapted from our previous study[39]. **(b)** Single-subject classification accuracy for each two-way classification and ROI. x axis: ROI; y axis: classification accuracy. Scattered dots and red lines represent the single-subject and group-level classification accuracies, respectively. Each color represents a different subject. Dashed lines represent the chance level (50%).

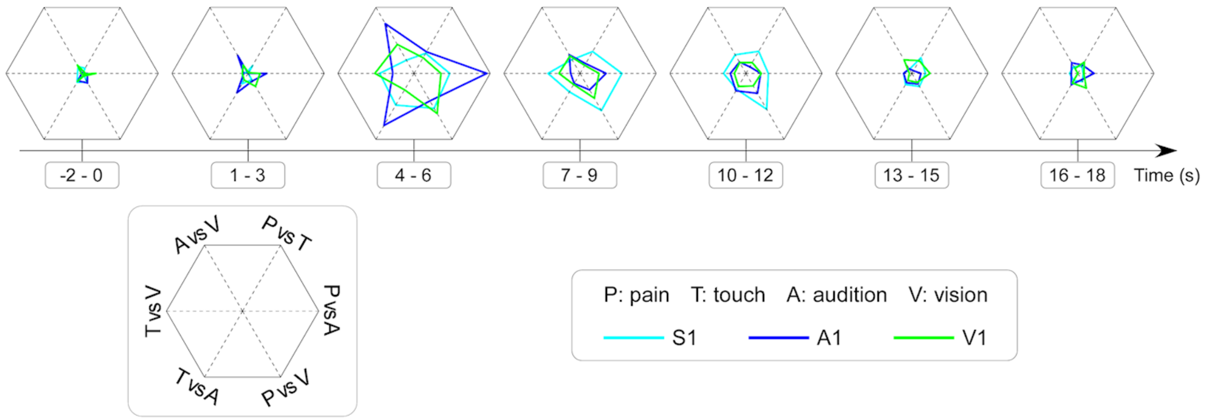

Supplementary Figure S2. Time-course of group-average classification accuracies, from -2s to +18s relative to stimulus onset. Each of the six axes of the radar plots indicates one of the six two-way classifications. Classification accuracies in each ROI are indicated in colours (S1: cyan; A1: blue; V1: green). The centre and the outer end of the axes represent chance-level (50%) and 80% accuracy, respectively.

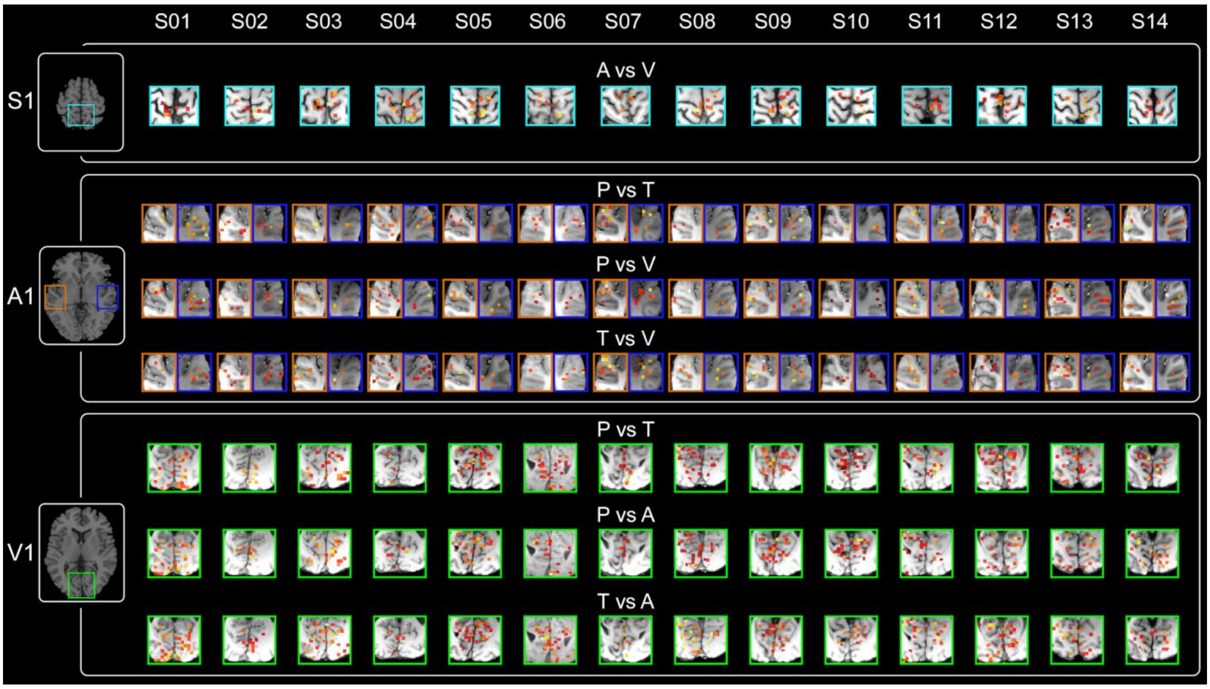

Supplementary Figure S3. Single-subject sensitivity maps of non-pertinent classification tasks. Maps are overlaid on individual structural scans normalized into MNI space. Each row represents a non-pertinent classification task. Each column represents a subject. SVM weights of the 20% of voxels contributing most to the classification (i.e. the 20% of voxels having the greatest SVM weights) are color-coded from red (lowest contribution) to yellow (highest contribution). Note that the most contributing voxels are widely scattered within each ROI. S1, primary somatosensory cortex. A1, primary auditory cortex. V1, primary visual cortex. P, pain. T, touch. A, audition. V, vision.

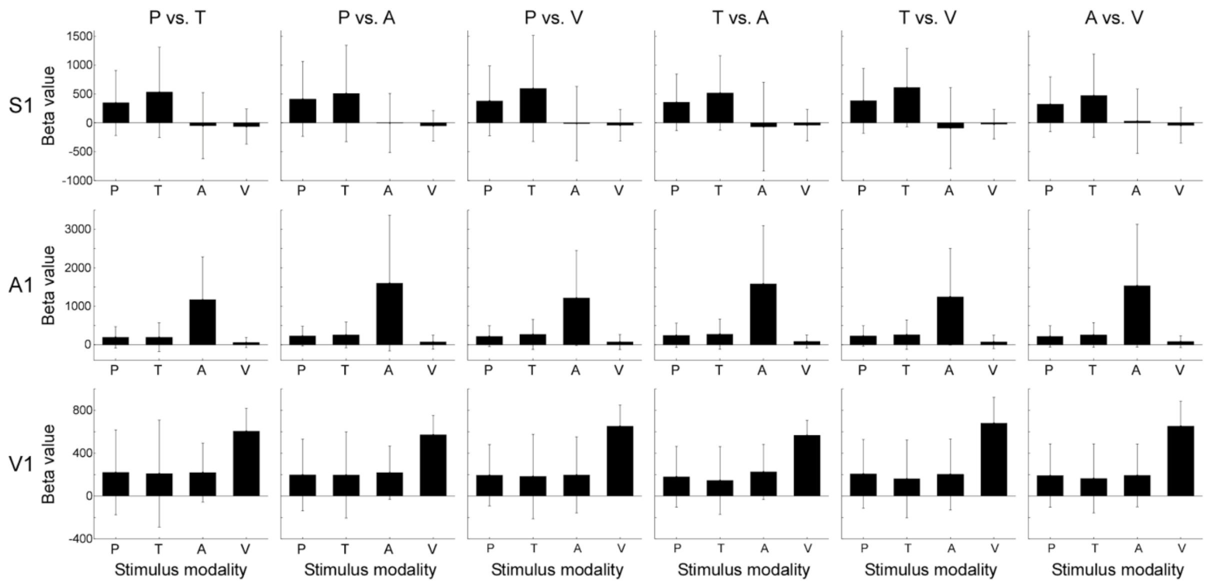

Supplementary Figure S4. Group-level mean and standard deviation of the average beta values of the 20% of voxels contributing most to each two-way classification, for each sensory modality. S1, primary somatosensory cortex; A1, primary auditory cortex; V1, primary visual cortex. P, pain; T, touch; A, audition; V, vision.

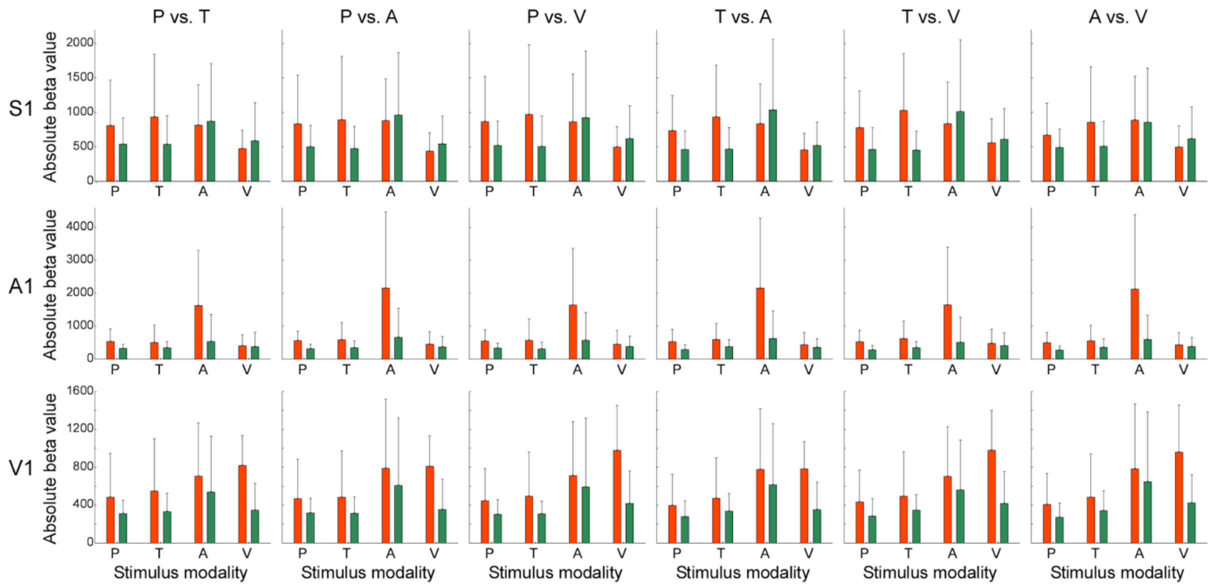

Supplementary Figure S5. Group-level mean and standard deviation of the average beta values of voxels showing positive (red) and negative (green) BOLD responses for each sensory modality in each sensitivity map. S1, primary somatosensory cortex; A1, primary auditory cortex; V1, primary visual cortex. P, pain; T, touch; A, audition; V, vision.

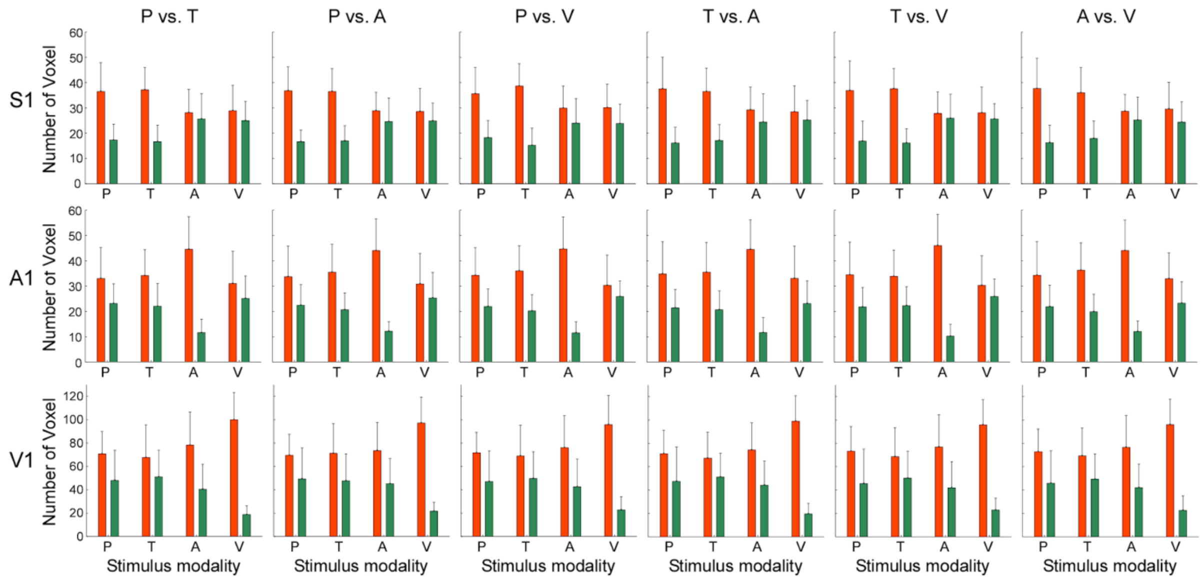

Supplementary Figure S6. Group-level mean and standard deviation of the number of voxels showing a positive (red) and negative (green) BOLD responses for each sensory modality in each sensitivity map. S1, primary somatosensory cortex; A1, primary auditory cortex; V1, primary visual cortex. P, pain; T, touch; A, audition; V, vision.

Control Analysis A – Eroded ROIs

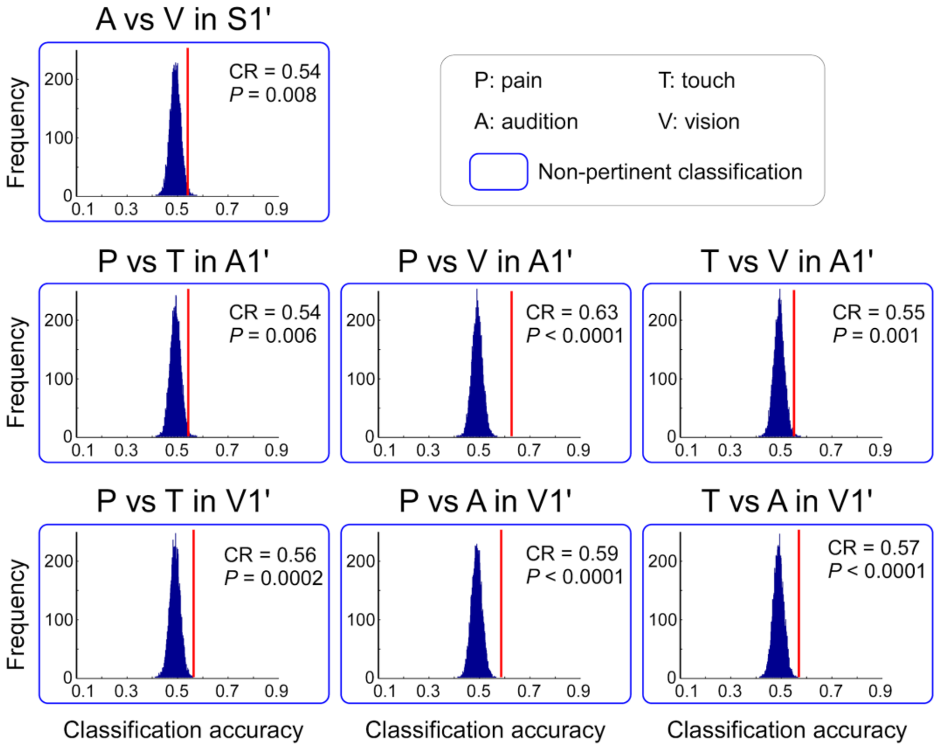

Supplementary Figure S7. Classification accuracies and null distributions of non-pertinent classifications, obtained using eroded ROIs. Group-average classification accuracies (CR, correct rate) and corresponding *P*-values obtained from 14 participants are shown in the top-right corner of each inset. Null distributions and *P*-values were generated using permutation test (*n*=10,000), as the main analysis. Given the substantially smaller number of voxels, the classification accuracies obtained using the eroded ROIs were, as expected, reduced. However, they were still clearly significantly higher than chance level in all non-pertinent classification tasks ($P < 0.01$). This result rules out the possibility that voxels located in the peripheral part of the primary sensory cortices, possibly sampling neural activity of neighbouring higher-order areas, determined the successful predictions. 'Non pertinent' classifications refer to the discrimination between two stimuli, neither of which corresponds to the principal modality of the ROI. S1', A1' and V1' labels denote eroded S1, A1 and V1 ROIs, respectively. P, pain. T, touch. A, audition. V, vision.

Control Analysis A – Manually defined ROIs

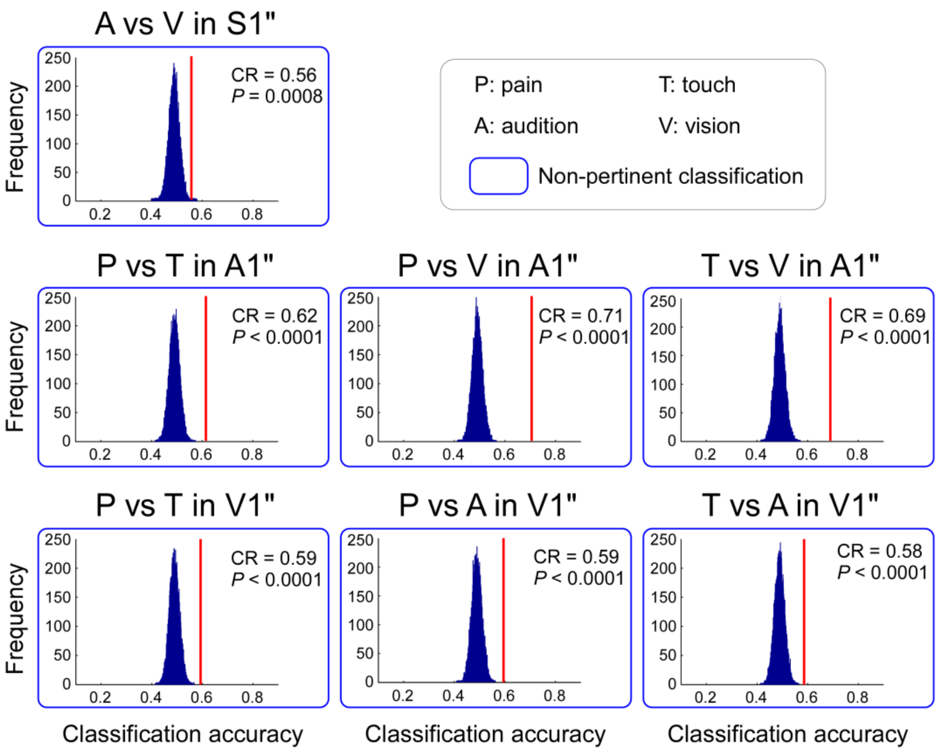

Supplementary Figure S8. Classification accuracies and null distributions of non-pertinent classifications, obtained using manually-defined ROIs. Group-average classification accuracies (CR, correct rate) and their corresponding *P*-values obtained from 14 participants are shown in the top-right corner of each inset. Null distributions and *P*-values were generated using permutation test (n=10,000), as the main analysis. Note that, using these manually-defined ROIs, classification accuracies are still significantly higher than chance level in all non-pertinent tasks ($P < 0.0001$). 'Non pertinent' classifications refer to the discrimination between two stimuli, neither of which corresponds to the principal modality of the ROI. S1", A1" and V1" labels denote manually-defined S1, A1 and V1 ROIs, respectively. P, pain. T, touch. A, audition. V, vision.

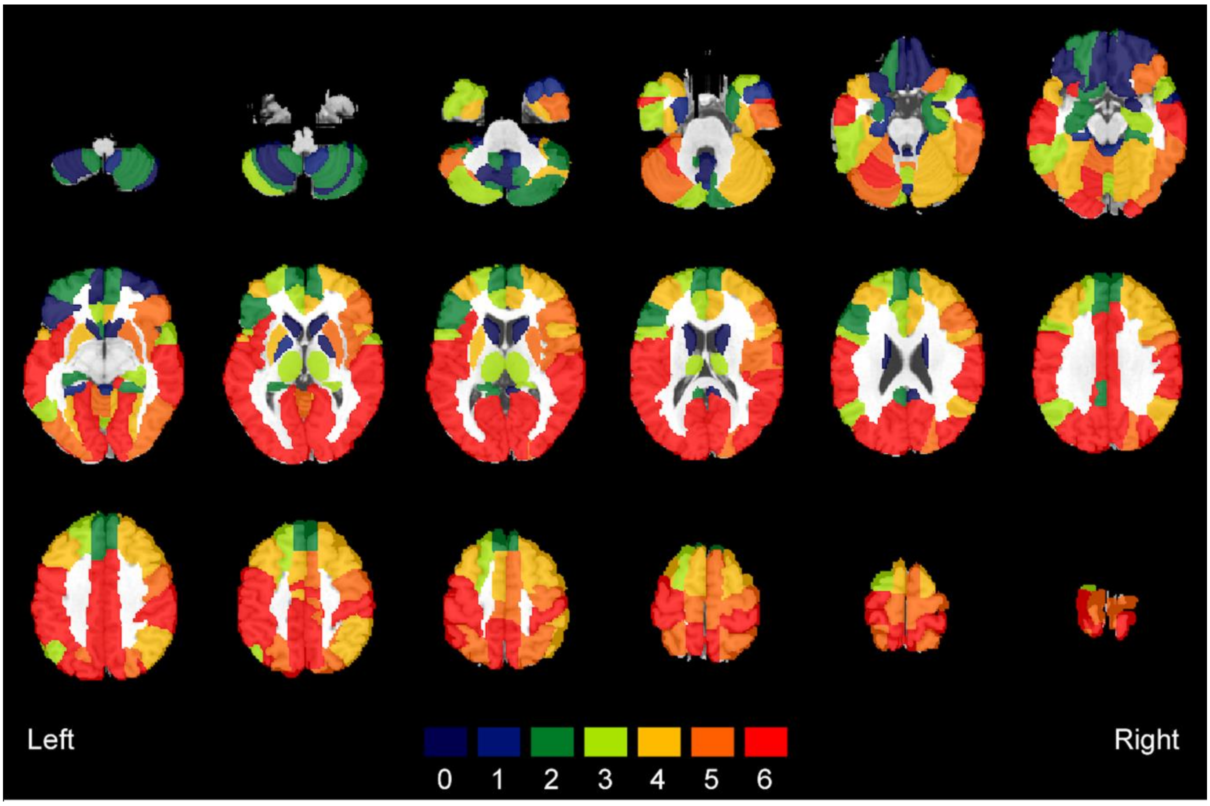

Supplementary Figure S9. Group-level results of whole-brain MVPA. Colours correspond to the number of classification tasks in which the signal from a given brain region allowed successful discrimination of stimuli of two different modalities. For example, if the signal of a given brain region yielded a classification accuracy significantly higher than chance level in all six classification tasks, that region was colour-coded in red. In this analysis, we obtained two main results. First, we confirmed that the patterns of BOLD signals elicited by stimuli of different sensory modalities in S1, A1 and V1 (defined using a different anatomical atlas: 'Postcentral gyri' for S1, 'Heschl gyri' for A1 and 'Calcarine fissure' for V1) are distinguishable (see also Supplementary Table S7). Second, when sorting the 116 brain regions constituting the whole brain according to their ability to predict the modality of the eliciting stimulus in the six two-way classification tasks, 24% were able to perform correctly in all six tasks, 16% in 5 tasks, 13% in 4 tasks, 10% in 3 tasks, 13% in 2 tasks, 7% in 1 task, and 17% in none of the six tasks (see also Supplementary Table S7). Hence, only a subset of all brain regions appears to contain information allowing discrimination of the sensory modality of the eliciting stimulus. This result also corroborates the results of the non-brain control ROI in the main analysis, by showing that there are brain structures whose BOLD signal does not seem to contain information about the sensory modality of the eliciting stimulus.

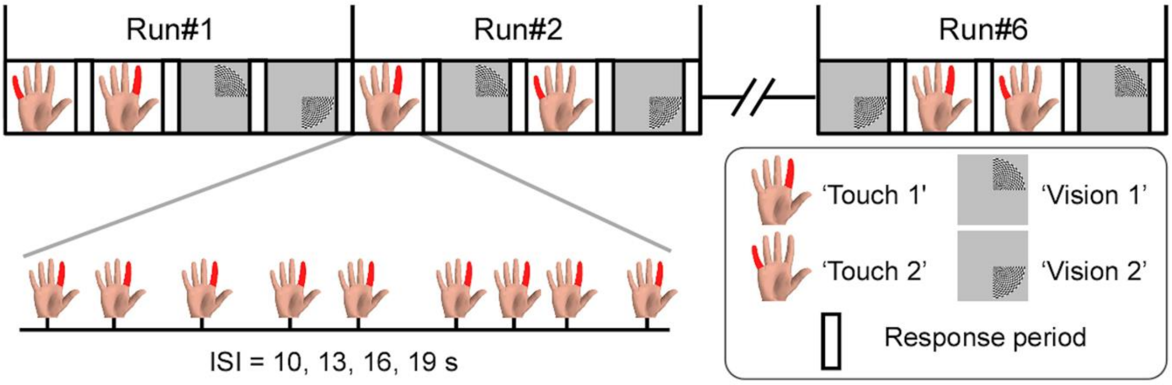

Supplementary Figure S10. Paradigm of Experiment 2. Sensory stimuli consisted in two different somatosensory stimuli (delivered to either the index finger, 'Touch 1', or the little finger, 'Touch 2') and two different visual stimuli (presented in either the upper-right visual field, 'Vision 1', or the lower-right visual field, 'Vision 2'). The fMRI session included six runs and each run consisted of four blocks. Each block contained only one type of stimuli and consisted of a stimulation period and a response period. During each stimulation period, 8 or 9 stimuli were presented with a pseudo-random inter-stimulus interval of 10, 13, 16, or 19 s. Participants were instructed to count the total number of stimuli perceived in each block. During the response period (duration = 30 s), two possible responses ("A: 8" or "B: 9") appeared on the screen, and participants had to indicate the number of stimuli perceived in this block by pressing one of two buttons with their left hand. Order of blocks was pseudo-randomized across runs within each subject.

Supplementary Table S1. Group level classification accuracies and corresponding *P*-values of each ROI for each two-way classification task.

	P vs. T (Mean±SD) (*P*-value)	P vs. A (Mean±SD) (*P*-value)	P vs. V (Mean±SD) (*P*-value)	T vs. A (Mean±SD) (*P*-value)	T vs. V (Mean±SD) (*P*-value)	A vs. V (Mean±SD) (*P*-value)	Average accuracy (Mean±SD)
**S1**	0.63 ± 0.13<br>*P* <.0001	0.68 ± 0.12<br>*P* <.0001	0.69 ± 0.14<br>*P* <.0001	0.67 ± 0.09<br>*P* <.0001	0.66 ± 0.08<br>*P* <.0001	0.58 ± 0.11<br>*P* <.0001	0.65 ± 0.07
**A1**	0.59 ± 0.09<br>*P* <.0001	0.83 ± 0.07<br>*P* <.0001	0.67 ± 0.08<br>*P* <.0001	0.78 ± 0.07<br>*P* <.0001	0.63 ± 0.07<br>*P* <.0001	0.78 ± 0.06<br>*P* <.0001	0.71 ± 0.04
**V1**	0.59 ± 0.10<br>*P* <.0001	0.61 ± 0.09<br>*P* <.0001	0.72 ± 0.12<br>*P* <.0001	0.57 ± 0.09<br>*P* <.0001	0.70 ± 0.13<br>*P* <.0001	0.68 ± 0.10<br>*P* <.0001	0.64 ± 0.06
**NB**	0.49 ± 0.09<br>*P* =.50	0.49 ± 0.08<br>*P* =.33	0.50 ± 0.09<br>*P* =.28	0.48 ± 0.08<br>*P* =.63	0.49 ± 0.08<br>*P* =.33	0.50 ± 0.06<br>*P* = .25	0.49 ± 0.04

Data presented as mean ± SD and p-values calculated using permutation testing, n=10,000

§P: pain, T: touch, A: audition; V: vision. S1: primary somatosensory cortex, A1: primary auditory cortex, V1: primary visual cortex, NB: non-brain region.

Supplementary Table S2. Statistical comparisons of classification accuracies between pertinent and non-pertinent classifications in A1.

		Non-pertinent classifications		
		P vs. T	P vs. V	T vs. V
Pertinent classifications	P vs. A	P = 6.10E-05	P = 6.10E-05	P = 6.10E-05
	T vs. A	P = 0.000244	P = 0.000854	P = 6.10E-05
	A vs. V	P = 0.000183	P = 0.001648	P = 0.000122

§Statistical significance is reported as p-values of non-parametric Wilcoxon signed-rank tests; P: pain; T: touch; A: audition; V: vision.

Supplementary Table S3. Statistical comparisons of classification accuracies between pertinent and non-pertinent classifications in V1.

		Non-pertinent classifications		
		P vs. T	P vs. A	T vs. A
Pertinent classifications	P vs. V	P = 0.000244	P = 0.005676	P = 0.004211
	T vs. V	P = 0.007935	P = 0.03009	P = 0.021973
	A vs. V	P = 0.012695	P = 0.025146	P = 0.015869

§Statistical significance is reported as p-values of non-parametric Wilcoxon signed-rank tests; P: pain; T: touch; A: audition; V: vision.

Supplementary Table S4. Statistical comparisons of classification accuracies between pertinent and non-pertinent classifications in S1.

		Non-pertinent classifications
		A vs. V
Pertinent classifications	P vs. T	P = 0.078552
	P vs. A	P = 0.043701
	P vs. V	P = 0.061768
	T vs. A	P = 0.015381
	T vs. V	P = 0.021606

§Statistical significance is reported as p-values of non-parametric Wilcoxon signed-rank tests; P: pain; T: touch; A: audition; V: vision.

Supplementary Table S5. Group-level classification accuraciesand corresponding *P* values obtained using signal from ipsilateral (R) and contralateral (L) ROIs.

		P vs. T (Mean±SD) (P value)	P vs. A (Mean±SD) (P value)	P vs. V (Mean±SD) (P value)	T vs. A (Mean±SD) (P value)	T vs. V (Mean±SD) (P value)	A vs. V (Mean±SD) (P value)
S1	L	0.62 ± 0.13 P <.0001	0.66 ± 0.13 P <.0001	0.67 ± 0.14 P <.0001	0.66 ± 0.10 P <.0001	0.66 ± 0.10 P <.0001	0.55 ± 0.12 P =.0007
	R	0.57 ± 0.12 P =.0001	0.62 ± 0.11 P <.0001	0.63 ± 0.11 P <.0001	0.63 ± 0.08 P <.0001	0.60 ± 0.06 P <.0001	0.57 ± 0.10 P =.0002
A1	L	0.59 ± 0.09 P <.0001	0.81 ± 0.08 P <.0001	0.66 ± 0.10 P <.0001	0.78 ± 0.09 P <.0001	0.61 ± 0.07 P <.0001	0.77 ± 0.07 P <.0001
	R	0.58 ± 0.11 P <.0001	0.75 ± 0.10 P <.0001	0.60 ± 0.10 P <.0001	0.73 ± 0.11 P <.0001	0.58 ± 0.10 P <.0001	0.70 ± 0.09 P <.0001
V1	L	0.58 ± 0.12 P <.0001	0.62 ± 0.09 P <.0001	0.73 ± 0.12 P <.0001	0.56 ± 0.09 P =.0001	0.69 ± 0.14 P <.0001	0.67 ± 0.11 P <.0001
	R	0.57 ± 0.11 P <.0001	0.60 ± 0.08 P <.0001	0.65 ± 0.14 P <.0001	0.56 ± 0.10 P =.0001	0.67 ± 0.12 P <.0001	0.64 ± 0.11 P <.0001

Data presented as mean ± SD

§ P: pain; T: touch; A: audition; V: vision.

Supplementary Table S6. Group-level classification accuracies and corresponding *P* values of each ROI for the four-way classification task.

	Pain (Mean±SD) (P value)	Touch (Mean±SD) (P value)	Audition (Mean±SD) (P value)	Vision (Mean±SD) (P value)	Average accuracy (Mean±SD)
**S1**	0.42 ± 0.21<br>P <.0001	0.47 ± 0.13<br>P <.0001	0.35 ± 0.10<br>P <.0001	0.38 ± 0.10<br>P <.0001	0.40 ± 0.08
**A1**	0.42 ± 0.15<br>P <.0001	0.41 ± 0.09<br>P <.0001	0.68 ± 0.11<br>P <.0001	0.45 ± 0.07<br>P <.0001	0.49 ± 0.06
**V1**	0.38 ± 0.16<br>P <.0001	0.38 ± 0.06<br>P <.0001	0.31 ± 0.11<br>P =.0013	0.50 ± 0.17<br>P <.0001	0.39 ± 0.08
**NB**	0.23 ± 0.08<br>P = 0.75	0.22 ± 0.08<br>P = 0.91	0.25 ± 0.06<br>P = 0.44	0.27 ± 0.09<br>P = 0.18	0.24 ± 0.05

Data presented as mean ± SD and and p-values calculated using permutation testing, n=10,000

§S1: primary somatosensory cortex, A1: primary auditory cortex, V1: primary visual cortex, NB: non-brain region.

Supplementary Table S7. Whole brain MVPA: group level classification accuracies (mean ± SD) of each of the 116 brain regions for each two-way classification task.

ROI	Classification accuracy (%)							N
	P vs. T	P vs. A	P vs. V	T vs. A	T vs. V	A vs. V	Mean accuracy	
Temporal_Sup_L	64.5 ± 9.8*	84.8 ± 9.1*	72.8 ± 8.6*	80.1 ± 11.4*	66.4 ± 11.0*	75.2 ± 10.5*	74.0 ± 7.8	6
Temporal_Sup_R	65.5 ± 12.0*	81.4 ± 8.2*	67.5 ± 10.2*	79.2 ± 8.3*	63.5 ± 8.3*	71.9 ± 12.1*	71.5 ± 7.4	6
Temporal_Mid_L	57.6 ± 13.7*	73.2 ± 11.7*	67.1 ± 11.3*	69.9 ± 9.6*	62.3 ± 11.3*	76.1 ± 10.1*	67.7 ± 6.9	6
Temporal_Mid_R	58.0 ± 11.7*	72.7 ± 11.6*	64.7 ± 10.9*	71.8 ± 6.6*	61.7 ± 10.5*	71.1 ± 11.4*	66.7 ± 6.1	6
Rolandic_Oper_L	62.4 ± 11.1*	70.5 ± 15.2*	69.2 ± 13.2*	68.5 ± 11.2*	65.8 ± 11.4*	61.8 ± 12.8*	66.4 ± 3.7	6
Insula_L	63.3 ± 11.8*	70.5 ± 13.4*	71.4 ± 12.7*	66.3 ± 8.4*	62.4 ± 11.2*	57.9 ± 10.2*	65.3 ± 5.2	6
Occipital_Mid_L	59.7 ± 12.1*	62.7 ± 8.3*	71.2 ± 9.3*	60.3 ± 8.3*	70.0 ± 10.4*	66.2 ± 9.9*	65.0 ± 4.9	6
Precuneus_L	58.4 ± 13.1*	64.8 ± 14.2*	70.4 ± 11.7*	65.4 ± 7.0*	68.6 ± 9.8*	58.7 ± 9.1*	64.4 ± 5.0	6
Cingulum_Mid_L	62.1 ± 13.7*	68.3 ± 13.9*	71.4 ± 15.5*	60.9 ± 9.6*	63.7 ± 7.2*	56.7 ± 14.3*	63.9 ± 5.3	6
Lingual_R	58.1 ± 10.2*	61.9 ± 8.3*	69.8 ± 13.0*	57.7 ± 9.2*	68.0 ± 12.1*	66.4 ± 12.5*	63.7 ± 5.1	6
Calcarine_L	59.9 ± 9.4*	62.4 ± 9.4*	70.0 ± 12.5*	59.7 ± 10.1*	63.7 ± 10.9*	65.7 ± 10.2*	63.6 ± 3.9	6
SupraMarginal_R	59.7 ± 9.5*	68.8 ± 8.7*	67.6 ± 8.4*	65.5 ± 7.4*	63.5 ± 7.9*	56.3 ± 9.5*	63.6 ± 4.8	6
SupraMarginal_L	60.0 ± 10.1*	67.0 ± 13.7*	70.9 ± 13.5*	63.4 ± 9.5*	61.7 ± 10.6*	57.4 ± 10.3*	63.4 ± 4.9	6
Lingual_L	56.5 ± 13.1*	62.5 ± 10.7*	68.4 ± 12.0*	56.0 ± 8.1*	67.4 ± 10.6*	66.4 ± 12.1*	62.9 ± 5.5	6
Cerebelum_6_L	58.8 ± 14.8*	61.9 ± 12.4*	70.1 ± 11.9*	57.9 ± 10.0*	65.2 ± 12.0*	63.2 ± 7.9*	62.9 ± 4.5	6
Cingulum_Mid_R	63.6 ± 10.7*	67.7 ± 11.5*	67.2 ± 12.0*	60.2 ± 11.2*	62.7 ± 8.6*	55.6 ± 10.6*	62.8 ± 4.5	6
Cuneus_R	57.9 ± 10.5*	61.0 ± 7.9*	70.2 ± 9.6*	59.7 ± 6.0*	66.1 ± 11.8*	61.6 ± 9.0*	62.8 ± 4.5	6
Postcentral_L	62.3 ± 13.3*	68.6 ± 12.7*	68.4 ± 11.9*	60.2 ± 9.0*	58.8 ± 9.4*	58.0 ± 11.0*	62.7 ± 4.7	6
Calcarine_R	57.8 ± 8.4*	59.4 ± 9.4*	67.0 ± 12.5*	58.5 ± 12.0*	68.1 ± 13.4*	65.1 ± 12.6*	62.6 ± 4.6	6
Occipital_Sup_L	56.5 ± 11.3*	62.7 ± 8.2*	68.5 ± 12.8*	59.4 ± 7.8*	64.8 ± 11.9*	63.8 ± 10.4*	62.6 ± 4.2	6
Precuneus_R	59.2 ± 11.2*	64.1 ± 12.2*	69.1 ± 10.6*	62.1 ± 6.9*	65.0 ± 8.4*	56.1 ± 10.3*	62.6 ± 4.6	6
Occipital_Mid_R	57.5 ± 11.4*	58.9 ± 7.1*	69.3 ± 9.5*	58.6 ± 7.6*	68.4 ± 6.1*	62.2 ± 8.6*	62.5 ± 5.2	6
Heschl_L	56.5 ± 9.7*	66.1 ± 12.7*	63.3 ± 12.2*	66.2 ± 10.2*	62.4 ± 5.5*	60.3 ± 10.9*	62.4 ± 3.7	6
Cuneus_L	58.4 ± 13.0*	60.0 ± 8.5*	70.2 ± 9.0*	58.0 ± 9.2*	64.8 ± 9.9*	62.1 ± 10.8*	62.3 ± 4.6	6
Heschl_R	57.4 ± 11.8*	68.6 ± 12.8*	67.2 ± 12.0*	60.8 ± 7.1*	59.5 ± 9.6*	55.7 ± 5.7*	61.5 ± 5.3	6
Precentral_L	61.4 ± 10.6*	65.8 ± 9.3*	65.0 ± 9.7*	58.7 ± 7.1*	58.0 ± 7.9*	59.5 ± 8.3*	61.4 ± 3.3	6
Parietal_Inf_L	57.6 ± 13.3*	61.9 ± 10.7*	68.0 ± 10.2*	58.4 ± 9.5*	62.1 ± 13.6*	57.9 ± 9.7*	61.0 ± 4.0	6
Postcentral_R	60.3 ± 14.1*	63.5 ± 11.5*	65.5 ± 10.8*	59.9 ± 10.6*	59.4 ± 6.4*	57.0 ± 8.8*	60.9 ± 3.1	6
Rolandic_Oper_R	63.7 ± 13.1*	73.5 ± 12.2*	67.6 ± 13.3*	62.5 ± 7.8*	64.7 ± 8.9*	54.9 ± 7.9	64.5 ± 6.1	5
Parietal_Sup_L	55.9 ± 11.6	62.2 ± 7.1*	68.6 ± 9.4*	61.0 ± 7.8*	70.3 ± 10.1*	63.2 ± 6.0*	63.5 ± 5.3	5
Insula_R	61.2 ± 8.5*	71.2 ± 11.8*	67.6 ± 11.3*	62.7 ± 9.2*	62.2 ± 9.8*	54.1 ± 10.2	63.2 ± 5.9	5
Occipital_Inf_L	53.2 ± 8.4	59.4 ± 7.4*	68.8 ± 9.1*	56.8 ± 7.0*	66.2 ± 13.2*	66.4 ± 8.8*	61.8 ± 6.2	5
Occipital_Inf_R	57.4 ± 11.9*	57.8 ± 11.6*	68.0 ± 8.8*	54.6 ± 7.1	65.0 ± 7.7*	62.1 ± 7.5*	60.8 ± 5.1	5

Precentral_R	61.0 ± 10.0*	63.6 ± 8.0*	65.3 ± 9.6*	54.6 ± 7.1	61.0 ± 7.9*	58.5 ± 9.1*	60.7 ± 3.8	5
Cerebelum_4_5_L	55.6 ± 11.3*	59.8 ± 11.3*	65.6 ± 12.6*	54.1 ± 9.3	63.2 ± 13.8*	63.4 ± 9.8*	60.3 ± 4.6	5
Parietal_Sup_R	53.7 ± 10.0	60.3 ± 9.1*	66.6 ± 10.3*	58.6 ± 7.9*	63.4 ± 8.7*	56.7 ± 8.2*	59.9 ± 4.7	5
Temporal_Inf_R	58.0 ± 10.8*	58.3 ± 10.7*	65.7 ± 10.5*	57.1 ± 8.3	59.3 ± 8.1*	59.6 ± 12.2*	59.7 ± 3.1	5
Occipital_Sup_R	52.3 ± 12.7	59.6 ± 5.3*	66.6 ± 7.7*	57.8 ± 8.9*	62.8 ± 8.1*	58.7 ± 12.7*	59.7 ± 4.8	5
Cerebelum_Crus1_L	57.7 ± 11.3*	57.7 ± 10.9*	64.5 ± 9.4*	55.7 ± 7.1	60.3 ± 8.9*	61.8 ± 11.1*	59.6 ± 3.2	5
Frontal_Inf_Tri_R	56.7 ± 9.6*	65.2 ± 10.7*	62.4 ± 12.0*	60.6 ± 10.9*	56.5 ± 8.8*	56.0 ± 10.0	59.6 ± 3.8	5
Putamen_R	59.6 ± 10.6*	64.3 ± 12.7*	62.2 ± 13.6*	59.0 ± 8.6*	57.4 ± 10.4*	53.3 ± 10.0	59.3 ± 3.8	5
Paracentral_Lobule_L	60.4 ± 12.5*	63.8 ± 13.7*	64.3 ± 12.8*	58.8 ± 7.7*	55.7 ± 8.5*	51.6 ± 9.5	59.1 ± 4.9	5
Supp_Motor_Area_R	56.6 ± 14.9*	62.9 ± 11.1*	64.8 ± 12.6*	58.8 ± 6.8*	56.1 ± 8.0	55.1 ± 9.1*	59.1 ± 4.0	5
Paracentral_Lobule_R	57.3 ± 13.0*	64.1 ± 11.1*	62.8 ± 14.4*	58.8 ± 8.1*	56.8 ± 10.5*	54.0 ± 7.8	59.0 ± 3.8	5
Vermis_4_5	57.7 ± 12.9*	59.0 ± 10.7*	62.8 ± 13.7*	55.4 ± 7.7	60.5 ± 10.7*	57.7 ± 8.6*	58.9 ± 2.6	5
Frontal_Inf_Orb_R	56.7 ± 9.4*	59.6 ± 9.5*	58.6 ± 9.0*	57.9 ± 11.5*	54.1 ± 9.3	57.5 ± 6.8*	57.4 ± 1.9	5
Cerebelum_6_R	54.9 ± 10.6	58.6 ± 8.8*	71.0 ± 9.2*	55.2 ± 10.4	66.4 ± 8.4*	62.3 ± 9.3*	61.4 ± 6.4	4
Fusiform_R	53.0 ± 9.4	58.1 ± 9.4*	70.1 ± 7.9*	52.9 ± 7.9	65.3 ± 8.4*	63.6 ± 8.0*	60.5 ± 7.0	4
Fusiform_L	55.9 ± 8.7	54.0 ± 6.3	66.6 ± 10.2*	56.4 ± 7.2*	65.3 ± 9.6*	63.2 ± 8.6*	60.2 ± 5.4	4
Frontal_Inf_Oper_R	59.7 ± 9.9*	64.3 ± 11.0*	63.4 ± 10.4*	53.9 ± 8.3	58.9 ± 9.4*	57.7 ± 8.3	59.7 ± 3.8	4
Cerebelum_4_5_R	54.0 ± 9.0	54.8 ± 11.5	67.1 ± 10.3*	55.7 ± 6.4*	63.3 ± 11.6*	60.4 ± 8.2*	59.2 ± 5.3	4
Cerebelum_Crus1_R	56.6 ± 10.2	58.1 ± 8.8*	64.4 ± 8.0*	53.9 ± 6.7	59.5 ± 7.3*	58.9 ± 10.4*	58.6 ± 3.5	4
Putamen_L	59.3 ± 14.0*	64.2 ± 13.3*	62.9 ± 13.8*	58.8 ± 8.4*	52.8 ± 10.0	51.8 ± 9.6	58.3 ± 5.1	4
Angular_R	57.4 ± 11.3*	57.5 ± 9.0*	62.6 ± 8.9*	55.2 ± 7.7	61.6 ± 6.2*	55.4 ± 12.5	58.3 ± 3.1	4
Frontal_Mid_R	60.3 ± 10.3*	59.7 ± 9.9*	62.5 ± 11.0*	57.0 ± 8.4*	56.8 ± 9.1	53.2 ± 10.6	58.3 ± 3.3	4
Temporal_Pole_Sup_L	55.6 ± 10.7	60.3 ± 10.1*	60.0 ± 11.2*	57.3 ± 6.6*	55.8 ± 9.5*	54.5 ± 8.7	57.2 ± 2.4	4
Parietal_Inf_R	52.9 ± 9.6	57.6 ± 9.0*	60.8 ± 13.6*	57.4 ± 7.2*	59.4 ± 6.4*	55.2 ± 10.0	57.2 ± 2.8	4
Frontal_Mid_L	58.4 ± 11.3*	62.1 ± 10.6*	59.8 ± 9.7*	56.1 ± 6.7*	53.5 ± 7.0	53.1 ± 10.0	57.2 ± 3.6	4
Cingulum_Ant_R	59.5 ± 9.2*	60.2 ± 10.9*	58.0 ± 8.9*	58.1 ± 10.5*	52.3 ± 9.6	52.2 ± 10.6	56.7 ± 3.5	4
Supp_Motor_Area_L	56.3 ± 9.7*	59.9 ± 11.2*	60.4 ± 12.1*	57.5 ± 8.7*	52.7 ± 11.2	52.6 ± 9.7	56.5 ± 3.4	4
Frontal_Sup_R	56.8 ± 11.3*	57.9 ± 9.9*	59.5 ± 9.6*	56.1 ± 7.8*	51.5 ± 6.5	51.9 ± 8.2	55.6 ± 3.3	4
Cingulum_Ant_L	58.1 ± 9.8*	61.9 ± 9.2*	60.0 ± 10.6*	56.0 ± 10.4	54.2 ± 10.1	53.3 ± 12.0	57.3 ± 3.4	3
Frontal_Inf_Oper_L	58.5 ± 11.8*	60.8 ± 11.9*	62.7 ± 12.0*	55.0 ± 9.5	53.9 ± 9.1	52.7 ± 6.0	57.3 ± 4.0	3
Temporal_Pole_Sup_R	54.1 ± 9.9	60.3 ± 10.6*	60.8 ± 11.5*	58.5 ± 9.2*	54.5 ± 7.8	54.6 ± 8.2	57.1 ± 3.1	3
Thalamus_L	55.5 ± 9.9	58.6 ± 11.1*	55.2 ± 12.6	59.6 ± 8.6*	50.7 ± 9.9	57.6 ± 12.1*	56.2 ± 3.2	3
Vermis_6	52.3 ± 9.7	60.0 ± 6.7*	59.8 ± 12.5*	52.2 ± 10.9	54.2 ± 8.7	58.0 ± 11.0*	56.1 ± 3.6	3
Frontal_Sup_L	57.4 ± 10.8*	60.4 ± 9.8*	57.7 ± 10.4*	53.2 ± 7.6	52.5 ± 5.2	54.6 ± 9.3	56.0 ± 3.0	3
Temporal_Inf_L	52.5 ± 10.0	54.9 ± 10.1	60.0 ± 8.4*	53.0 ± 7.2	57.4 ± 10.8*	57.1 ± 10.8*	55.8 ± 2.9	3
Hippocampus_R	56.6 ± 8.5*	57.9 ± 10.0*	58.4 ± 11.3*	53.9 ± 7.4	53.5 ± 7.1	53.8 ± 8.3	55.7 ± 2.2	3
Thalamus_R	53.7 ± 12.1	58.7 ± 11.3*	59.5 ± 9.9*	54.6 ± 10.7	50.9 ± 10.8	56.3 ± 8.6*	55.6 ± 3.2	3

Angular_L	55.5 ± 12.2*	55.8 ± 10.4*	59.8 ± 11.9*	52.1 ± 9.4	54.8 ± 7.6	55.1 ± 10.1	55.5 ± 2.5	3
Temporal_Pole_Mid_L	53.3 ± 8.2	54.1 ± 8.7	56.8 ± 7.0*	53.1 ± 8.0	56.5 ± 7.0*	56.3 ± 7.9*	55.0 ± 1.7	3
Cerebelum_Crus2_L	56.7 ± 12.2*	58.7 ± 9.0*	56.9 ± 8.7*	52.7 ± 10.2	49.2 ± 8.2	53.5 ± 8.3	54.6 ± 3.5	3
Frontal_Inf_Tri_L	56.7 ± 7.5	60.3 ± 9.3*	61.6 ± 11.0*	54.6 ± 9.6	52.5 ± 11.2	53.3 ± 8.8	56.5 ± 3.8	2
Frontal_Sup_Medial_R	55.5 ± 6.2	58.8 ± 10.3*	57.8 ± 8.0*	54.9 ± 8.4	52.7 ± 6.9	55.1 ± 10.0	55.8 ± 2.2	2
Hippocampus_L	55.7 ± 13.6	55.0 ± 12.0	61.0 ± 10.6*	56.7 ± 7.2*	50.8 ± 7.4	53.6 ± 8.0	55.5 ± 3.4	2
Frontal_Mid_Orb_R	57.1 ± 9.2*	57.5 ± 8.6*	54.9 ± 8.4	55.9 ± 6.7	51.1 ± 6.1	55.4 ± 6.5	55.3 ± 2.3	2
Cerebelum_8_R	55.9 ± 10.9	59.7 ± 6.7*	57.4 ± 8.0*	55.8 ± 4.8	51.3 ± 5.2	51.6 ± 9.2	55.3 ± 3.3	2
Amygdala_L	54.5 ± 8.6	57.0 ± 7.4*	58.8 ± 12.9*	54.6 ± 7.9	52.2 ± 9.3	53.9 ± 10.7	55.2 ± 2.4	2
Cingulum_Post_L	53.0 ± 12.1	56.0 ± 12.5*	55.5 ± 8.1	57.6 ± 6.2*	50.8 ± 7.8	55.2 ± 9.4	54.7 ± 2.4	2
Frontal_Sup_Orb_L	55.7 ± 7.2	57.1 ± 6.1*	55.4 ± 7.5	56.7 ± 4.2*	51.3 ± 7.9	51.3 ± 6.8	54.6 ± 2.6	2
Frontal_Sup_Medial_L	55.6 ± 7.0	58.3 ± 8.8*	56.4 ± 8.4*	53.3 ± 8.0	49.8 ± 9.7	54.2 ± 9.0	54.6 ± 2.9	2
Cerebelum_Crus2_R	53.8 ± 7.3	58.7 ± 8.5*	58.1 ± 9.8*	52.8 ± 8.6	50.4 ± 7.6	53.2 ± 10.3	54.5 ± 3.2	2
ParaHippocampal_R	53.9 ± 11.9	55.9 ± 10.5*	59.6 ± 9.5*	51.0 ± 8.6	52.1 ± 10.4	52.6 ± 6.5	54.2 ± 3.1	2
Cerebelum_9_L	53.6 ± 9.2	55.6 ± 6.9*	56.8 ± 8.6*	52.2 ± 8.9	52.0 ± 8.2	54.7 ± 8.8	54.1 ± 1.9	2
Frontal_Mid_Orb_L	53.7 ± 8.1	56.4 ± 9.2*	55.0 ± 10.6	55.1 ± 10.4*	52.3 ± 9.4	49.2 ± 7.5	53.6 ± 2.6	2
Cerebelum_7b_L	56.8 ± 8.5*	54.5 ± 9.6	57.4 ± 8.3*	49.8 ± 7.7	51.5 ± 6.8	51.8 ± 8.4	53.6 ± 3.1	2
Olfactory_L	55.8 ± 7.7*	49.8 ± 4.3	57.4 ± 8.6*	49.4 ± 8.9	50.8 ± 8.5	50.8 ± 8.6	52.3 ± 3.4	2
Pallidum_L	55.2 ± 12.8	55.2 ± 12.3	55.0 ± 12.5	55.5 ± 7.2*	53.8 ± 4.7	53.9 ± 10.5	54.8 ± 0.7	1
Cerebelum_3_R	55.1 ± 7.6	54.2 ± 6.7	54.1 ± 9.0	52.0 ± 6.9	51.0 ± 10.6	56.3 ± 8.7*	53.8 ± 2.0	1
Temporal_Pole_Mid_R	51.5 ± 12.6	52.7 ± 8.7	57.1 ± 11.3*	55.2 ± 8.9	53.6 ± 7.3	51.8 ± 6.6	53.6 ± 2.2	1
ParaHippocampal_L	51.3 ± 11.5	53.0 ± 12.2	56.1 ± 8.3*	51.5 ± 10.4	52.7 ± 8.4	53.8 ± 10.1	53.1 ± 1.8	1
Vermis_7	54.5 ± 9.4	56.3 ± 8.1*	52.8 ± 7.3	53.1 ± 8.3	50.1 ± 9.3	51.7 ± 8.8	53.1 ± 2.1	1
Vermis_3	55.1 ± 9.2	56.4 ± 7.4*	54.2 ± 10.1	50.2 ± 9.8	49.7 ± 7.2	52.2 ± 11.6	53.0 ± 2.7	1
Cerebelum_9_R	49.2 ± 9.4	55.5 ± 7.5	57.4 ± 8.6*	51.3 ± 7.8	53.5 ± 11.6	50.8 ± 7.6	52.9 ± 3.1	1
Cerebelum_10_L	50.6 ± 7.3	52.5 ± 7.0	56.3 ± 10.6*	48.3 ± 6.3	51.9 ± 8.9	49.1 ± 8.5	51.4 ± 2.8	1
Frontal_Mid_Orb_R	55.4 ± 7.5	55.6 ± 11.4	56.6 ± 9.4	53.6 ± 7.5	54.4 ± 5.0	52.3 ± 6.9	54.6 ± 1.5	0
Frontal_Inf_Orb_L	55.2 ± 6.5	54.8 ± 9.1	55.6 ± 7.5	55.1 ± 9.7	50.9 ± 8.1	52.9 ± 10.5	54.1 ± 1.8	0
Cingulum_Post_R	54.7 ± 8.7	51.5 ± 8.8	53.2 ± 9.3	53.2 ± 8.8	54.1 ± 4.7	55.1 ± 9.4	53.6 ± 1.3	0
Rectus_L	53.1 ± 7.6	52.8 ± 7.2	53.9 ± 8.5	54.6 ± 7.2	53.8 ± 6.5	52.5 ± 7.7	53.4 ± 0.8	0
Caudate_L	50.8 ± 11.8	53.7 ± 7.8	54.9 ± 10.0	53.6 ± 10.7	51.6 ± 7.9	53.7 ± 7.2	53.0 ± 1.5	0
Pallidum_R	54.9 ± 10.9	54.9 ± 9.7	54.5 ± 8.3	52.9 ± 6.3	48.2 ± 5.3	52.0 ± 7.8	52.9 ± 2.6	0
Cerebelum_8_L	51.7 ± 11.1	54.7 ± 8.3	55.5 ± 11.9	52.5 ± 11.3	49.8 ± 7.7	52.7 ± 9.6	52.8 ± 2.1	0
Frontal_Sup_Orb_R	54.2 ± 8.2	54.5 ± 6.6	53.7 ± 12.1	53.1 ± 7.8	50.4 ± 7.7	50.1 ± 5.0	52.7 ± 1.9	0
Caudate_R	52.7 ± 7.3	55.8 ± 7.5	50.4 ± 7.6	51.7 ± 10.2	52.7 ± 6.8	52.8 ± 9.5	52.7 ± 1.8	0
Rectus_R	53.0 ± 7.8	51.7 ± 7.3	54.5 ± 6.5	53.0 ± 5.7	51.7 ± 4.7	51.3 ± 8.1	52.5 ± 1.2	0
Amygdala_R	53.7 ± 11.0	53.9 ± 10.6	54.6 ± 8.6	51.5 ± 11.7	51.7 ± 9.7	49.9 ± 10.1	52.5 ± 1.8	0

Cerebelum_10_R	52.1 ± 9.3	54.1 ± 9.0	56.0 ± 9.4	49.9 ± 10.0	50.3 ± 7.0	52.2 ± 8.9	52.5 ± 2.3	0
Cerebelum_7b_R	49.6 ± 7.2	52.3 ± 7.6	54.0 ± 8.1	51.7 ± 7.4	52.6 ± 7.4	54.1 ± 7.7	52.4 ± 1.7	0
Frontal_Mid_Orb_L	49.0 ± 5.7	54.9 ± 5.4	51.2 ± 5.1	55.1 ± 8.5	50.2 ± 7.5	52.3 ± 8.6	52.1 ± 2.5	0
Cerebelum_3_L	53.2 ± 7.0	52.9 ± 8.3	51.5 ± 8.3	49.1 ± 9.4	53.3 ± 8.9	52.7 ± 8.6	52.1 ± 1.6	0
Vermis_8	50.8 ± 10.0	53.0 ± 8.5	51.2 ± 7.0	51.7 ± 6.9	52.2 ± 7.2	51.1 ± 8.2	51.7 ± 0.8	0
Vermis_9	53.6 ± 7.5	51.2 ± 7.0	52.7 ± 6.4	50.4 ± 7.6	51.9 ± 8.1	47.9 ± 7.1	51.3 ± 2.0	0
Vermis_10	48.9 ± 7.2	52.9 ± 7.3	51.0 ± 8.9	50.7 ± 7.1	49.0 ± 9.5	53.0 ± 6.2	50.9 ± 1.8	0
Olfactory_R	51.7 ± 5.9	53.1 ± 7.4	51.8 ± 6.7	50.7 ± 7.1	49.4 ± 6.5	48.2 ± 6.8	50.8 ± 1.8	0
Vermis_1_2	50.3 ± 6.7	48.5 ± 6.1	49.2 ± 7.2	48.0 ± 5.3	49.7 ± 4.4	49.6 ± 5.2	49.2 ± 0.8	0

[§]P: pain; T: touch; A: audition; V: vision. N: number of tasks in which the signal from a given brain region allowed successful discrimination of stimuli of two different modalities. Classification accuracies that are significantly higher than chance level ($P < 0.05$, corrected) are denoted by asterisks (*). The order of the regions are sorted firstly by the value of N and then by the value of Mean accuracy.

Supplementary Methods

Experiment 1

Participants

Fourteen healthy right-handed volunteers took part in the study (6 females, aged 20-36 years). All participants gave written informed consent, and the experimental procedures were approved by the Oxford Central University Research Ethics Committee (CUREC).

Sensory stimuli

Lights in the scanner room were dim. While lying in the scanner, participants received stimuli of four different sensory modalities: touch, pain, audition and vision. All stimuli were delivered to or around the participant's right lower limb[39]. Nociceptive somatosensory (painful) stimuli were pulses of radiant heat (5 ms duration) generated by an infrared neodymium yttrium aluminium perovskite (Nd:YAP) laser (wavelength: 1.34 μm; ElEn Group, Italy). The laser beam was transmitted through an optic fibre, and focusing lenses were used to set its diameter at target site to ~7 mm. The energy of the stimulus (3 ± 0.5 J) was set to elicit a clear painful pinprick sensation, related to the selective activation of Aδ skin nociceptors, without the contribution of tactile afferents[46]. The stimulus was applied to the dorsum of the right foot, within the sensory territory of the superficial peroneal nerve. To prevent fatigue of sensitisation of nociceptors, the laser beam was manually displaced by ~2 cm after each stimulus. Non-nociceptive somatosensory (non-painful tactile) stimuli were constant current square-wave electrical pulses (1 ms duration; DS7A, Digitimer Ltd, UK), delivered through a pair of skin electrodes (1 cm inter-electrode distance) placed at the right ankle, over the superficial peroneal nerve. For each participant, stimulus intensity (6 ± 2 mA) was adjusted to elicit a non-painful paresthesia in the sensory territory of the nerve. The intensity of electrical stimulation was above the electrical activation threshold of Aβ fibres (which convey innocuous non-nociceptive sensations) but well below the electrical activation threshold of nociceptive Aδ and C fibres[47,48], and

never elicited a painful percept. Visual stimuli consisted of a bright white disk (~9° viewing angle) displayed on the projection screen, above the right foot, for 100 ms. Auditory stimuli were loud (65 dB), right-lateralised 800 Hz tones (0.5 left/right amplitude ratio; 50 ms duration; 5 ms rise and fall times), delivered binaurally through custom-built pneumatic earphones bored into a set of low-profile ear defenders[49].

Experimental paradigm

The fMRI experiment consisted of a single acquisition, divided into four runs. Each run consisted of a stimulation period (~8 min duration), followed by a rating period (~2 min duration). During the stimulation period, each type of stimulus was delivered 8 times (4 stimulus modalities x 8 = 32 stimuli/period). All stimuli were delivered in a pseudo-random order, such that stimuli of the same sensory modality were not delivered consecutively more than twice. The sequence of stimulation was different in each run. The inter-stimulus interval (ISI) was 10, 13, 16 or 19 s. For each stimulus modality, each ISI was used 8 times (4 stimulus modalities x 4 ISI x 8 = 128 stimuli in total). The order of ISIs was pseudo-randomised, such that the same ISI was not used consecutively more than twice. Throughout the stimulation sequence, participants were instructed to fixate a white cross (~1.5° viewing angle) displayed at the centre of the screen. During the rating period, participants were asked to rate the saliency of each type of stimulus. This was done by adjusting the position of a cursor on four consecutively-displayed visual-analogue scales, labelled “laser”, “electric”, “visual” and “auditory”. Each scale was displayed for 9 s. For each rating, the position of the cursor was transformed into a numerical value between 0 and 10. Left and right extremities of the scales were labelled “not salient” and “extremely salient”. The order of presentation of the four scales was randomised across blocks. Stimulus saliency was explained to each participant as “the ability of the stimulus to capture attention”[40]. Therefore, this behavioural feedback was expected to integrate several factors such as stimulus intensity, frequency of appearance, novelty and its potential relevance to behaviour. Several studies have shown that human judgments of saliency correlate well

with predicted models of saliency[41]. The average ratings of saliency were not significantly different across modalities (repeated measures ANOVA: $F_{(3,39)}$ =0.75, P =0.53).

Imaging data acquisition

Functional MRI data were acquired using a 3T Varian-Siemens whole body scanner (Oxford Magnet Technology, UK). A head-only gradient coil was used with a birdcage radiofrequency coil for pulse transmission and signal reception (whole-brain gradient-echo time; 41 contiguous 3.5 mm-thick slices; field of view 192x192 mm; matrix 64x64; repetition time (TR) of 3 s; 740 volumes, resulting in a total scan time of 37 minutes). At the end of the experiment, a T1-weighted structural image (1 mm-thick axial slices, in-plane resolution 1x1 mm) was acquired for spatial registration and anatomical overlay of the functional data.

Data pre-processing

The first four fMRI volumes were discarded to allow for signal equilibration. The remaining volumes were spatially aligned to the middle volume to correct for head movements using the motion correction tool (MCFLIRT) implemented in FSL (www.fmrib.ox.ac.uk/fsl). The aligned fMRI volumes were further detrended using head motion parameters estimated during the spatial alignment, to remove the possible effect of head motion on BOLD signal, and were linear detrended within each fMRI run to remove possible signal drift over time. Detrending was performed using the software package PyMVPA (www.pymvpa.org/index.html)[45]. To test whether head motion was different following the delivery of the different types of stimuli, for each subject we performed a one-way ANOVA across the four stimulus conditions (four levels: pain, touch, audition and vision), for each of the six head motion parameters (three translations and three rotations) during the acquisition of the 2nd volume after stimulus onset (i.e., the volume used in the main MVPA analysis). In all subjects, none of the six parameters showed significant differences across conditions (P_{min}=0.39, P_{max}=1.00, P_{median}=0.97). In order to preserve all spatial information and prevent any possible unwanted effect

induced by data pre-processing (such as signal spreading over space and time), the BOLD signals were not spatially smoothed or temporally filtered.

To avoid possible confounds of non-specific differences between stimuli such as differences in saliency, arousal or attention, which may result in differences in average intensity of activation in each given ROI under different stimulus conditions, fMRI data were normalised, for each ROI, by (1) subtracting from the signal value at each time point of each voxel the mean signal value across all voxels of the ROI at the same time point, and (2) dividing the result by the standard deviation of the signal from all voxels of the ROI at that time point. After such normalisation procedure, at each time point, the group of voxels constituting the ROI had a mean of zero and a standard deviation of 1. Thus, after this normalisation procedure, within each ROI, the BOLD signals recorded under the different stimulation conditions differed only in terms of their spatial distribution.

A brief introduction of multivariate pattern analysis (MVPA)

Multivariate pattern analysis (MVPA) is a machine learning technique that uses a pattern classifier[21,43] to identify the representational content of the neural responses elicited by different stimuli. Unlike conventional mass-univariate analyses such as the general linear model (GLM), which detects regional-average activation and only consider one single voxel at a time, MVPA detects patterns of activity across many voxels, and, thereby, infers that a specific representation of the stimulus is contained in the spatial pattern of activity sampled across multiple voxels of a given brain region[21]. Therefore, MVPA is more sensitive than conventional univariate analysis in disclosing differences in brain activities between experimental conditions, as it may detect changes in the spatial distribution of BOLD signals even when regional-average activity does not differ across different conditions[44]. MVPA often takes the form of solving a classification problem. For example, the whole fMRI dataset obtained in an experiment is divided into a training dataset and a test dataset. The training dataset, together with the known labels (identifying the different stimulus categories or experimental conditions), is used to train a classifier that learns the pattern of

responses to each stimulus category. This classifier is then applied to the test dataset to assess its ability to predict the modality of the stimulus eliciting the responses of each sample. A good classification accuracy implies that the data (i.e. the spatial distribution of the BOLD signal within a given brain region) contains sufficient information to distinguish correctly the different stimulus categories or experimental conditions.

Permutation test

To test whether the accuracy of the classifier was higher than chance level (i.e., 0.5 for two-way classifications and 0.25 for four-way classifications), we built the null distributions of the classification accuracies under the scenario in which each fMRI volume is labelled randomly and thus does not contain any information about the modality of the stimulus eliciting the recorded fMRI response during training of the classifier. Such permutation test (n=10,000) was performed for each classification task, ROI and subject, and for both training and testing datasets (similar results were observed when permuting training dataset only). From each of these single-subject null distributions, a group-level null distribution was subsequently generated by randomly selecting one data point from each single-subject null distribution, averaging them, and repeating this procedure (random selection followed by averaging) 10,000 times. Finally, by comparing the classification accuracy obtained from the correctly-labelled dataset with the group-level null distributions, we obtained a non-parametric P-value expressing the significance of their difference, for each classification task and ROI. Such permutation test was performed for both two-way and four-way classifications.

Control analyses

Control analysis A. The ROIs defining the primary sensory cortices, generated using the Jülich probabilistic atlas[19], were transformed into each single subject's space and then trimmed individually to ensure accurate anatomical position of each ROI. However, it could be argued that the successful predictions of the seven non-pertinent classifications could be merely determined by the fMRI signal

obtained from voxels included in the primary sensory cortices ROIs, but possibly belonging to neighbouring higher-level areas. Although this explanation is unlikely, given the widely scattered distribution of the voxels contributing to the classification accuracy (Fig. 2a and Supplementary Fig. S3), two additional control analyses were performed to test this possibility.

In the first analysis, we repeated the MVPA on smaller, eroded ROIs, generated by removing the voxels constituting the outer layer of the original ROIs. The original ROIs were first resampled to a resolution of 2x2x2 mm^3, and then the first outer layer of voxels (defined as voxels having at least one neighbouring voxel not belonging to the original ROIs) was removed. After resampling the ROIs to their original 3x3x3 mm^3 resolution, this procedure resulted in eroded ROIs with, on average, 39±9%, 29±5% and 46±5% of the total number of voxels constituting the original S1, A1 and V1 ROIs. The same MVPA and statistical analyses were then performed such as in the main analysis.

To provide an additional control, we also manually defined ROIs corresponding to S1 (foot area), A1 and V1 on the high-resolution structural image of each participant, and transformed them into the corresponding functional image (a procedure that we used in previous studies[50]). The same MVPA and statistical analyses were then performed such as in the main analysis.

Control analysis B. The results obtained in the main analysis are highly hypothesis-driven, that is, we aimed to test whether primary sensory cortices contain a distinguishable spatial pattern of BOLD signals elicited by stimuli of their non-corresponding sensory modalities. Given that different sensory modalities can be discriminated using BOLD signals sampled from primary sensory cortices, in Control analysis B we tested whether this feature is pervasive across the whole brain. The brain was parcellated into 116 regions based on the AAL atlas[23]. These 116 regions were initially transformed to each individual space to account for the individual variability. The same MVPA (two-way classifications) and permutation testing were performed on each of these regions using the same procedure of the main analysis with the exception of the following two differences: (1) the number of permutation was 2,000 instead of 10,000, to reduce the computational time due to the large

number of ROIs; (2) as the performance of 6 classification tasks was evaluated in 116 regions, the *P*-values were corrected for multiple comparisons (i.e., $P < 0.05/116/6 = 0.00007$).

Experiment 2

Functional MRI data were collected from a different group of fourteen healthy participants (21-26 years, mean ±SD = 22.6 ±1.7 years; 7 females) who gave written informed consent and the experimental procedures were approved by the Southwest University Ethics Committee. Participants received two different somatosensory stimuli and two different visual stimuli. Somatosensory stimuli were 10-Hz innocuous electrical pulses (1-ms duration for each pulse) lasting for 200 ms, delivered to either the index finger ('Touch 1') or the little finger ('Touch 2') of the right hand using ring electrodes. Visual stimuli were 10-Hz pattern reversal wedge-shaped checkerboards of 90° arc lasting for 200 ms, and delivered to either the upper-right ('Vision 1') or the lower-right ('Vision 2') visual field.

Functional MRI data were acquired using a 3T General Electric whole body scanner, with a resolution of 3x3x3.5 mm^3 and a TR of 3 s. The experimental design is represented in Supplementary Fig. S10. Briefly, fMRI data were collected in a single session divided in six runs. Each run consisted of four blocks. Each block contained only one type of stimulus and consisted of a stimulation period and a response period. An interval of ~2 minutes was inserted between fMRI runs, to reduce the fatigue and movement of subjects during the stimulation period. During each stimulation period, 8 or 9 stimuli were presented with a pseudo-random inter-stimulus interval of 10, 13, 16, or 19 s. Participants were instructed to fixate a white point at the centre of the screen, pay attention to the stimulation and count the total number of stimuli. During the response period (duration = 30 s), two possible responses ("A: 8" or "B: 9") appeared on the screen, and participants had to indicate the number of stimuli perceived in this block by pressing one of two buttons with their left hand (overall response accuracy across subjects: Mean±SD = 89.3±10.9%). The order of the blocks was pseudo-randomized across runs within each subject. A T1-weighted structural image (1x1x1 mm^3) was

acquired for spatial registration and anatomical overlay of functional data. The same acquisition parameters for fMRI and structural images as Experiment 1 were used.

The same preprocessing steps of Experiment 1 were used. The primary sensory cortices ROIs (S1, A1 and V1) were created for each individual brain using the Jülich probability atlas. A leave-two-runs-out cross-validation was used for the following two-way classification tasks: (1) 'Touch 1' vs. 'Touch 2' using BOLD signals in V1; (2) 'Vision 1' vs. 'Vision 2' using BOLD signals in S1; (3) 'Touch 1' vs. 'Touch 2' using BOLD signals in A1; (4) 'Vision 1' vs. 'Vision 2' using BOLD signals in A1; (5) 'Touch' vs. 'Vision' using BOLD signals in A1. For the classification task (5), 'Touch 1' and 'Touch 2' stimuli were pooled together as 'Touch' stimuli, and similarly, 'Vision 1' and 'Vision 2' stimuli were pooled together as 'Vision' stimuli. These five classification tasks were chosen to test (1) whether the BOLD signal in primary sensory cortices allows discriminating between stimuli of the same non-corresponding modality presented at different locations of the receptive surface, and (2) whether successful classification could be observed when stimuli of the same modality but different spatial features were pooled.

Supplementary References

46 Bromm, B. & Treede, R. D. Nerve fibre discharges, cerebral potentials and sensations induced by CO2 laser stimulation. *Hum Neurobiol* **3**, 33-40 (1984).

47 Burgess, P. R. & Perl, E. R. Myelinated afferent fibres responding specifically to noxious stimulation of the skin. *J Physiol* **190**, 541-562 (1967).

48 Mouraux, A., Iannetti, G. D. & Plaghki, L. Low intensity intra-epidermal electrical stimulation can activate Adelta-nociceptors selectively. *Pain* **150**, 199-207 (2010).

49 Mayhew, S. D., Dirckx, S. G., Niazy, R. K., Iannetti, G. D. & Wise, R. G. EEG signatures of auditory activity correlate with simultaneously recorded fMRI responses in humans. *Neuroimage* **49**, 849-864 (2010).

50 Iannetti, G. D. *et al.* Pharmacological modulation of pain-related brain activity during normal and central sensitization states in humans. *Proc Natl Acad Sci U S A* **102**, 18195-18200 (2005).
